# Assessment of Graphical Methods for Determination of the Limiting Current Density in Complex Electrodialysis-Feed Solutions

**DOI:** 10.3390/membranes12020241

**Published:** 2022-02-18

**Authors:** Katarina Knežević, Daniela Reif, Michael Harasek, Jörg Krampe, Norbert Kreuzinger

**Affiliations:** 1 Institute for Water Quality and Resource Management, TU Wien, 1040 Vienna, Austria; dreif@iwag.tuwien.ac.at (D.R.); jkrampe@iwag.tuwien.ac.at (J.K.); norbkreu@iwag.tuwien.ac.at (N.K.); 2 Institute of Chemical, Environmental and Bioscience Engineering, TU Wien, 1060 Vienna, Austria; michael.harasek@tuwien.ac.at

**Keywords:** electrodialysis, limiting current density, wastewater treatment, nutrient recovery

## Abstract

Electrodialysis (ED) is a promising technology suitable for nutrient recovery from a wide variety of liquid waste streams. For optimal operating conditions, the limiting current density (LCD) has to be determined separately for each treated feed and ED equipment. LCD is most frequently assessed in the NaCl solutions. In this paper, five graphical methods available in literature were reviewed for LCD determination in a series of five feed solutions with different levels of complexity in ion and matrix composition. Wastewater from microbial fermentation was included among the feed solutions, containing charged and uncharged particles. The experiments, running in the batch ED with an online conductivity, temperature, and pH monitoring, were conducted to obtain data for the comparison of various LCD determination methods. The results revealed complements and divergences between the applied LCD methods with increasing feed concentrations and composition complexity. The Cowan and Brown method had the most consistent results for all of the feed solutions. Online conductivity monitoring was linearly correlated with the decreasing ion concentration in the feed solution and corresponding LCD. Therefore, the results obtained in this study can be applied as a base for the automatized dynamic control of the operating current density–voltage in the batch ED. Conductivity alone should not be used for the ED control since LCD depends on the ion exchange membranes, feed flow, temperature and concentration, ionic species, their concentration ratios, and uncharged particles of the feed solution.

## 1. Introduction

Electrodialysis (ED) is an electro-membrane ion separation process widely used to demineralize, concentrate, and/or convert salt-containing solutions [1,2,3,4]. Moreover, ED appears as an emerging technology for nutrient recovery in wastewater treatment [5,6,7,8]. The driving force for the salt separation is the application of an electrical potential across an ED membrane stack, whereby charged particles are removed from the feed solution. An ED membrane stack comprises alternately placed cation- and anion-exchange membranes. During ED, the feed stream is separated into two streams, one with a reduced concentration of ions (diluate), and the other enriched with ions (concentrate). From the techno-economical point of view, it is important to run ED under optimal operating conditions [9,10,11,12]. Various parameters, such as feed solution composition, temperature, flow, membrane characteristics, spacers, applied power, and current density, impact the ED process efficiency [2,13,14,15,16,17,18]. One of the core parameters for the ED process design is the limiting current density (LCD) [19,20,21,22,23]. The experimentally determined LCD is commonly used for deriving the process essential boundary layer thickness, and it is directly proportional to the ion concentration and diffusivity [20,22,24]. The boundary layer is a thin layer of the solution adjacent to the membrane surface, with a concentration decrease of counter-ions on the diluate side and a concentration increase of co-ions on the concentrate side, compared to the bulk concentration [16]. The LCD indicates the electric current density that leads to the depletion of ions in the boundary layer on the diluate side, as a result of the concentration polarization [24]. With a further increase of the current density above a certain threshold, a dissociation of water molecules to H^+^ and OH^−^ ions occurs in the boundary layer as an undesired effect, causing an increase of the overall current density and subsequently a decrease in the current efficiency. Two additional transport mechanisms, gravitational convection and electroconvection, were proven to significantly affect the generation of current in the over limiting conditions [2,21,25,26,27]. The LCD increases with the increasing flow, temperature, and ion concentration of the solution [16,28,29,30,31].

The LCD values are difficult to predict for complex solutions, such as municipal or industrial wastewater, and must be determined in advance for each treated feed solution in lab-scale experiments. In the past, batch ED processes had been mostly operated under constant current conditions, which were chosen based on the LCD of the targeted diluate concentration. However, recent publications propose a dynamic current density control, dependent on the actual diluate compositions and ED stack characteristics [32,33]. Considerable research focused on defining the boundary layer formation, resolving the problems it causes and determining the LCD in advance for optimal process design and operation [19,20,22,34,35,36]. Graphical methods are most commonly suggested in the literature to determine the LCD and corresponding operating current density [2,16,19,35,37,38,39,40]. However, the obtained results are often contradictory. Furthermore, most of the developed methods were conducted with simplified matrices as sodium chloride solutions and rarely with "real-life" media. Since ED is emerging as an interesting method for nutrient recovery from various liquid waste streams, there is a need to assess the applicability of the existing LCD determination methods on complex feed solutions.

This paper assessed the applicability of five established LCD graphical methods from the literature to feed solutions with increasing grades of complexity. The considered graphical methods are Isaacson and Sonin [15,37], Cowan and Brown [35], the pH method [38], a method based on the current efficiency (λ) proposed by La Cerva et al. [19], and on the desalting efficiency (ε) proposed by Meng et al. [36]. The feed solution complexity investigated in this paper was divided into three levels: (a) Single-salt (NaCl, Na_2_SO_4_) solutions, (b) complex synthetic media (various ions added in changing ratios), and (c) real liquid waste streams from a microbial fermentation process. All of the LCD experiments were performed with the same ED set-up in the batch mode, only varying the feed matrix. The diluate concentration decreases continuously during the ED ion separation process in the batch mode. Consequently, the LCD changes as well, leading to the requirements for a consecutive adaptation of the operating conditions. Therefore, the concentration ranges were assessed on the LCD, and the online conductivity measurement, as a direct correlation to the ionic concentration, was applied for the monitoring. The experimental data were analyzed using the methods mentioned above. This study aimed at answering the following questions: (I) Are the applied methods for the LCD determination equally applicable to a wide range of salt concentrations; (II) Are the methods equally applicable to each type of feed representing an increasing complexity; (III) Do the solutions with the same conductivity but different ionic composition and level of complexity, reach the same LCD; (IV) Is there a potential for using a simple measurement, such as the conductivity, for a quick LCD determination of simple and complex synthetic media as well as real complex solutions.

## 2. Materials and Methods

### 2.1. Experimental Set-Up

A 10-cell pair lab-scale ED 64-004 unit (PCCell GmbH, Heusweiler, Germany) was used for the experimental work. The utilized membranes were 10 anion-exchange membranes (AEM, PC SA) and nine cation-exchange membranes (CEM, PC SK) from the same producer, with an active membrane area of 8 × 8 cm^2^ per membrane. The membrane characteristics are in Table 1, and the relative contribution of the membranes to the whole ED stack resistance is in Supplementary Material. Polypropylene spacers (PCCell) had a thickness of 0.45 mm. Both of the end membranes were set up as cation-exchange membranes to minimize the chloride ion transport towards the electrodes. Pt/Ir-coated titanium anode and V4A steel cathode were placed in the polypropylene electrode housing material. A DC power supply was applied for adjusting the constant current–voltage operating mode. The two double-wall feed tanks (2 L), one for the diluate and one for the concentrate, were filled with identical feed solutions at the beginning of each experiment. Cooling water was circulated through the outer layer of the double-wall tanks, maintaining the feed temperature at a constant level. Online measurements of the conductivity, temperature, and pH of the diluate outlet, and the conductivity and temperature (JUMO GmbH and Co., Fulda, Germany) of the concentrate outlet were measured every 2 s. Operational data were automatically recorded in a csv-file via the data acquisition box, as originally supplied by the producer.

**Figure 1:**
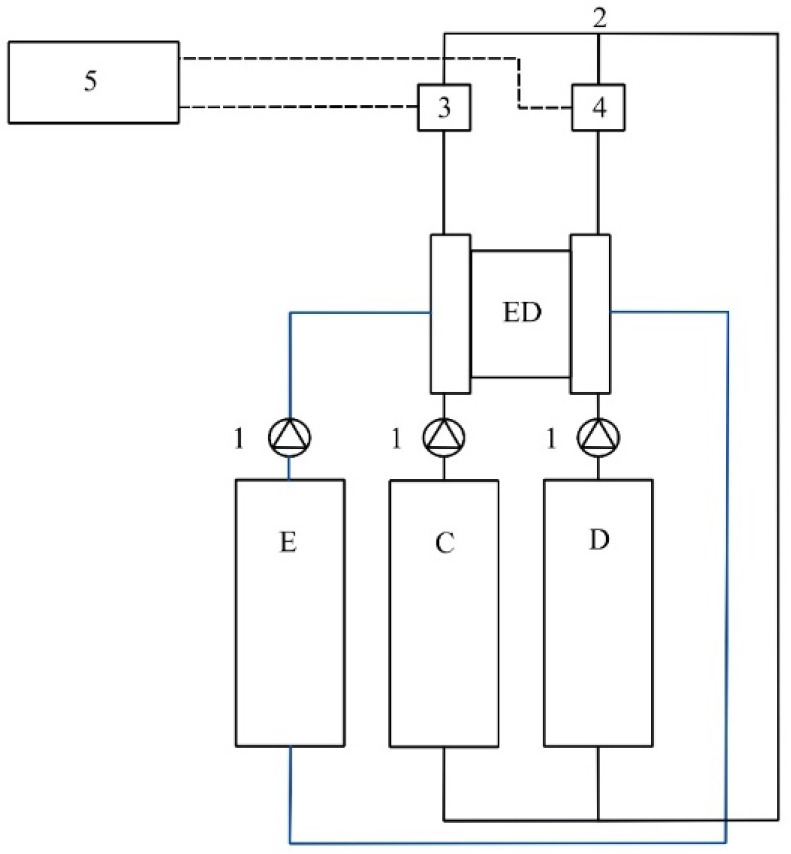
A schematic representation of the used experimental set-up, including the ED membrane stack (ED), concentrate (C), diluate (D), electrode rinse (E), centrifugal pumps (1), mixing pipe (2), conductivity and temperature sensors (3), conductivity, temperature, and pH sensors (4), data acquisition box (5).

The ED unit was operated in a batch mode and the operating conditions could be adjusted only manually. For all of the performed LCD tests, both of the obtained streams, the diluate and the concentrate stream, were continuously mixed again after the membrane stack to maintain the feed concentration at a constant level during the experiments (Figure 1). Flows of the diluate and concentrate solutions were maintained with centrifugal pumps (ITS-Betzel, Hatterscheim, Germany) by applying a constant flow rate of 15 L/h, with an average velocity of 0.012 m/s in each diluate/concentrate chamber. A 0.25 M Na_2_SO_4_ (5 L) electrolyte solution was circulated between the electrodes with a flow rate of 150 L/h. The applied voltage was increased stepwise from 3–5 V to a maximum of 29 V, in 0.5–1 V increments.

### 2.2. Description of Assessed LCD Determination Methods

Commonly, the feed stream circulates inside the diluate chamber in the ED batch mode until the desired demineralization rate is reached. This indicates that the diluate salt concentration, as well as its conductivity, are continuously decreasing. Since the salt concentration directly impacts the LCD, the lowest current density should be applied throughout the ED desalting process or the current density should be gradually reduced. Therefore, the ranges of the salt concentrations were subjected to a diapason of current densities (voltages) and analyzed via graphical LCD methods.

Various measured parameters, such as current density, voltage, conductivity, and pH, can indicate the properties and behavior of the treated solution. Several authors correlated these parameters to lead to knowledge in the ED. Therefore, the LCD has been defined as a crucial parameter for understanding the treated solution behavior and ED process control. The applied graphical LCD methods are described in the following sections, and their graphical presentations can be found in Appendix A.

#### 2.2.1. Isaacson and Sonin (I–S) Method

**Figure A1:**
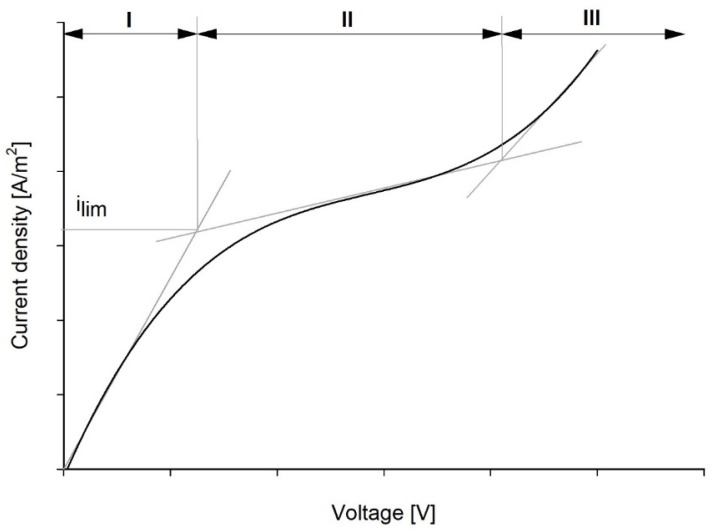
Current–voltage curve for an ED stack, showing LCDs of an electrolyte solution according to the Isaacson and Sonin method [37].

Isaacson and Sonin (I–S) method analyzes the current density (mA/cm2) behavior depending on the voltage applied to the ED cell [37]. Three different regions (I–III) are defined by this method (Appendix A, Figure A1). (I) is the Ohmic region with a linear correlation of the current density with the applied voltage. (II) is a region where the linear dependence starts leveling out. Here, the operational mode is transfiguring to the limiting current density region, indicating an energy loss due to the increased voltage resulting in the dissociation of water molecules to H^+^ and OH^−^ ions, gravitational convection due to density differences, and electroconvection [21,41]. This occurs due to the boundary layer formation adjacent to the membrane surface on the diluate side. The intersection point of the two slopes belonging to the Ohmic and the second (plateau) region indicates the LCD. One way to extend the Ohmic region would be to increase the feed flow, allowing for better mixing of the ions present in the treated solution and reduction of near-membrane boundary layer formation [42]. The third region (III) extends over the increasing positive slope, where a second intersection point can be determined. This region can be explained by the contribution of already split water molecules to the conducting electricity through the ED cell, gravitational convection, electroconvection, and the current carried by the salt counter-ions.

In this research, the head and the tail of the experimental data were fitted by a linear equation, as presented in Appendix A, Figure A1. The LCD was found at their intersecting point, calculated as:

(1) $$y\left(i_{lim}\right)=m_{1}\cdot \frac{b_{2}-b_{1}}{m_{1}-m_{2}}+b_{1}$$

where the *m_1_* and *m_2_* are the slopes of the first and second fitting equation, respectively, and *b_1_* and *b_2_* are the intercepts of the first and second fitting equation, respectively.

#### 2.2.2. Cowan and Brown (C–B) Method

**Figure A2:**
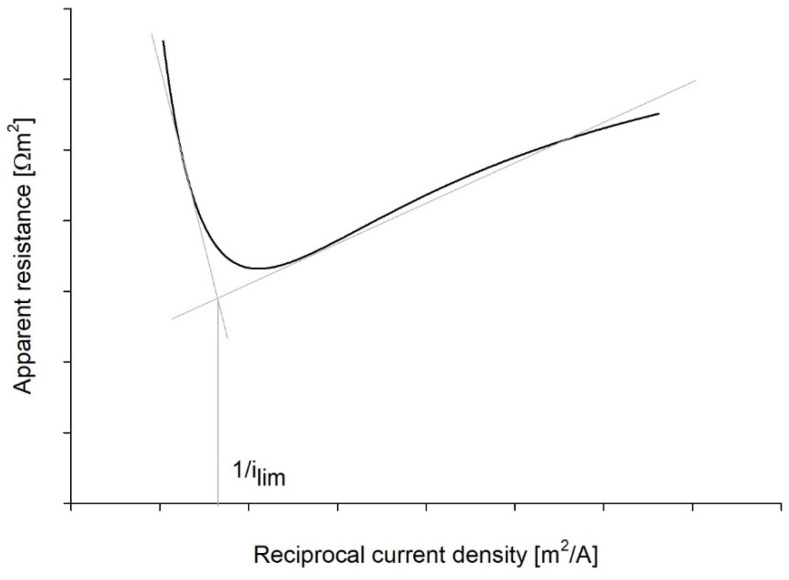
LCD assessed according to the Cowan and Brown method [35], correlating the reciprocal current density and ED stack resistance.

Cowan and Brown (C–B) [35] presented their data differently than Isaacson and Sonin. Rather than analyzing a direct current density–voltage dependency, the ED cell resistance is plotted against the reciprocal current density (Appendix A, Figure A2). LCD manifests at a polarization voltage as a rapid change of the slope. The ED cell resistance is decreasing with the increase of the current density up to a certain point. At this point, with a further increase of the current density (applied voltage), regions with higher resistance in the ED cell evolve, drawing a negative slope on the diagram. The intersection of the positive and negative slope represents the LCD. In this study, the experimental data were fitted by an inverse second-order polynomial function, and the LCD is determined as the function minimum:

(2) $$f\left(x\right)=y_{0}-\frac{a}{x}+\frac{b}{x^{2}}$$

Values of the function coefficients (*y_0_*, *a* and *b*), *R^2^*, intersection of the slopes, and current density (1/*x*) for minimum resistance can be found in Supplementary Table S2.

#### 2.2.3. The pH Method

**Figure A3:**
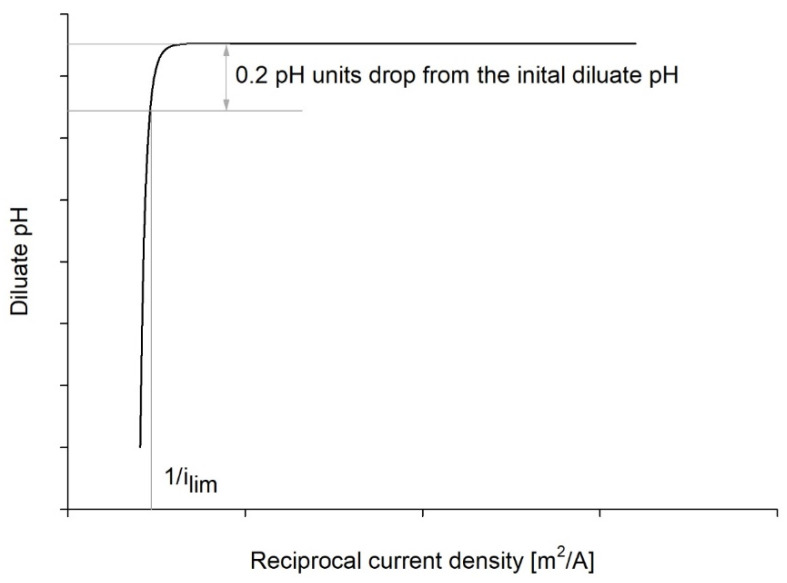
LCD determined as the sudden pH drop of the diluate solution with the increased current density [38].

Cowan and Brown noticed that the pH of the diluting stream starts to decrease at a current value very close to the value at which the resistance slope changes [35]. Consequently, the authors [38] define the limiting current density for the pH method as a point where the pH value of the diluting stream shows a decrease of 0.2 pH units. This breaking point can be seen on the diluate pH–reciprocal current density diagram (Appendix A, Figure A3). However, the initial pH of the tested feed solutions varies strongly, and in some of the experiments it actually increases with the increasing current density. Therefore, *ΔpH* was introduced to compare the pH trends among the solutions with different levels of complexity, calculated as follows:

(3) $$\Delta pH=pH_{dil}^{OUT}-pH_{dil}^{IN}=-log\frac{{\left[H^{+}\right]}_{t}}{{\left[H^{+}\right]}_{0}}\;\;\;\;\;\;\;\;$$

where the subscripts *pH_dil_^IN^* and *pH_dil_^OUT^* are the pH values of the diluate inlet and outlet, respectively. When the diluate pH drops, the H^+^ ion concentration increases and *ΔpH* is <0, and vice versa.

#### 2.2.4. Current Efficiency (λ) Method

**Figure A4:**
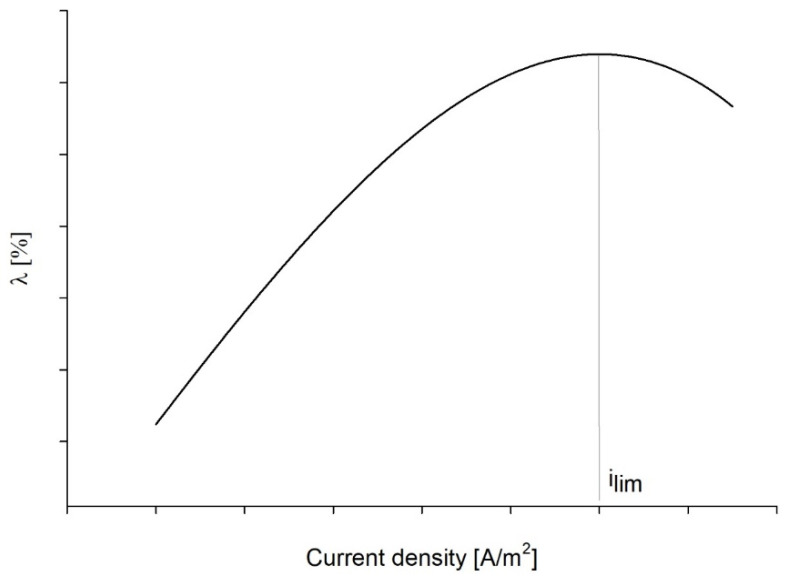
LCD determined as the maximum current efficiency, according to the method proposed by La Cerva et al. [19].

La Cerva et al. [19] examined the research of Kwak et al. [43] and suggested plotting the current efficiency (*λ*) against the current density, wherein the LCD appears at the maximum *λ* (%) (Appendix A, Figure A4). This method is an alternative to the desalting efficiency method proposed by Meng et al. [36] for determining the optimal operating current. The following equation was used to calculate *λ*:

(4) $$\lambda =\frac{zFQ_{dil}\left(C_{dil}^{IN}-C_{dil}^{OUT}\right)}{NI}\;\cdot 100\%$$

where *z* is the ionic charge, *F* is Faraday constant (96485.33 sA/mol), *Q_dil_* is the flow of the diluate stream (L/s), *C_dil_^IN^* and *C_dil_^OUT^* are the inlet and outlet concentrations of the diluate stream (mol/L), *I* is the current value (A), and *N* is the number of the ED cell pairs. NaCl concentrations are calculated based on the NaCl concentration–conductivity correlation (Supplementary, Figure S2).

#### 2.2.5. Desalting Efficiency (ε) Method

**Figure A5:**
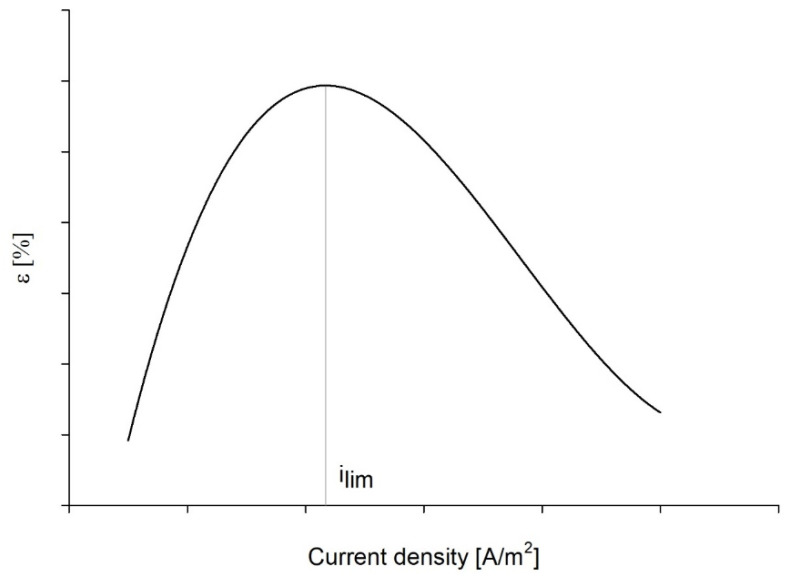
LCD appearing at the maximum desalting efficiency, according to the method proposed by Meng et al. [36].

Meng et al. [36] proposed a method for determining the optimal operating current based on the desalting efficiency (ε):

(5) $$\varepsilon =\frac{S_{dil}^{IN}-\kappa_{dil}^{OUT}}{\kappa_{dil}^{IN}}\;\cdot 100\%$$

where *κ_dil_^IN^* is the initial conductivity (mS/cm) of the feed to be treated, and *κ_dil_^OUT^* is the conductivity (mS/cm) of diluate generated by the ED. Values of the desalting efficiency are plotted against the current density, wherein the LCD appears as the maximum *ε* (%), representing the optimal operating current (Appendix A, Figure A5).

In this research, the *ε* values were plotted against the voltage, since the experimental data had similar trends to the C–B curves. Therefore, the head and tail of the experimental data were fitted by a linear equation and the LCD was found at their intersecting point. The ε–i plots can be found in Supplementary Material.

### 2.3. Solution Tested

To assess the five methods for determining the LCD, a set of experimental ED runs with different synthetic and real feed media were performed. All of the reagents used in this study were purchased from Merck KGaA (Darmstadt, Germany). The pH values of the synthetically prepared solutions are rather low (~5) due to the absorption of atmospheric carbon dioxide and the production of carbonic acid in the deionized water used for the NaCl solution preparation [43].

#### 2.3.1. Solution Level a—Single-Salt Solution (SSS)

Analytical grade NaCl was added to the deionized water to meet the concentration ranges between the surface waters and seawater in five concentrations between 0.005 and 0.5 M. The corresponding measured electrical conductivities ranged from 1–48.7 mS/cm, respectively. In further experiments, 0.03 M NaCl solution with a conductivity of 5.5 mS/cm was assigned as SSS1 (see Table 2) for comparison to the other analyzed solutions. In the next approach, analytical grade Na_2_SO_4_ (SSS2) was added to the deionized water to meet the conductivity of 5.5 mS/cm and to compare its behavior during the LCD experiments to the other treated solutions (see Table 2). Sulfate-containing salt was chosen due to the high SO_4_^2−^ content in the real complex solutions investigated in a later step (see Section 2.3.3). Additionally, the impact of the ionic radii and ionic mobility on the LCD can be studied when NaCl and Na_2_SO_4_ solutions with the same conductivity (ionic strengths) are subjected to the same conditions for the LCD assessment.

#### 2.3.2. Solution Level b—Synthetic Complex Solution (SCS)

Two model solutions based on the ionic content of the waste streams from two different fermentation processes (SCS1 and SCS2) without any organic matter content were prepared with the analytical grade salts (see Table 2). SCS1 (5.5 mS/cm) solution has already been studied in a previous research [44]. Organic compounds, commonly present in real waste streams, were not added to these model solutions in order to avoid their interference in LCD determination. Dilution ranges from the initial model solutions (SCS1 and SCS2) were chosen and prepared for the LCD assessment, keeping in mind the decreasing LCD with decreasing salt content in the diluate stream during the batch ED. The following conductivities for the dilution steps used as feed solutions were obtained: 2.9, 1.4, and 0.2 mS/cm for the SCS1 serial; and 7.7, 5.3, 2.8, and 1.9 mS/cm for the SCS2 serial. The pH value of the SCS2 was adjusted to 3 by adding 20% of sulfuric acid.

#### 2.3.3. Solution Level c—Real Complex Solution (RCS)—Real Liquid Waste Streams

As macro- and micro-nutrients are usually added to fermentation media in excess, their unused residuals remain in the liquid waste at the end of the process. There is a high potential to recover these elements via ED and reuse them in a new fermentation process. As an example for this application, a real waste stream from fermentation with *Sulfolobus acidocaldarius* was used and analyzed in dilution ranges [45,46]. *S. acidocaldarius* is recognized as a key organism in future biotechnology due to its multiple commercial products [47]. The initial characteristics of the fermentation media (RCS) are shown in Table 2. The fermentation effluent was microfiltered before the ED application as a barrier to residual bacterial cells. The waste streams of the fermentation processes often vary in salt concentration. Therefore, the dilution ranges were selected to meet specific conductivities (11.2, 7.5, 5.1, 2.7, 1.9 mS/cm).

### 2.4. Data Processing

R Studio (version 3.6.1) was used for data processing. An R-script was written, taking the ED unit’s text-file output as a script input, processing data based on the five LCD assessment methods, and displaying LCD values for each method and media used. Numeric data were consolidated by diagrammatic visualization of the data.

## 3. Results and Discussion

Results of experimental LCD tests are categorized into three sections. First, graphical LCD methods were proven for the feed NaCl dilution ranges. Second, LCD was assessed for feed solutions with the different ionic complexity, but the same initial conductivity. Third, one LCD method was selected for the LCD–conductivity correlation between the solutions with varying ionic complexity and inlet conductivity.

### 3.1. SSS—Range of NaCl Concentrations

Starting from a simple NaCl solution, five LCD assessment methods were applied to five increasing NaCl concentrations and compared to each other (LCD values in Table 3). LCD was mostly studied in the ED application for seawater desalination. Therefore, ion transport mechanisms, such as migration and diffusion, along with the occurring phenomena appearing, such as osmosis and electro-osmosis, in the limiting and over limiting regions are available in literature [16,21,48]. LCD is expected to be seen in a flattening of the I–U curve (I–S), sudden ED stack resistance increase (C–B), sudden diluate pH drop (pH-method), maximum point in the current efficiency (λ), and desalting efficiency (ε) followed by a sudden drop of the current/desalting efficiency.

**Figure 2:**
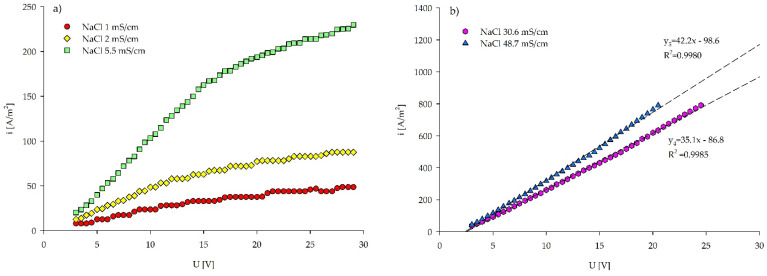
Current–voltage curves based on the Isaacson and Sonin method for estimating LCD in (**a**) three lower concentrated NaCl solutions and (**b**) two higher concentrated NaCl solutions. The detailed image of the fitting curves and their precision in three less concentrated NaCl solutions can be found in Supplementary Figure S3 and Table S1.

Overall slopes increased with the increasing salt concentrations, since the current density increases with the increasing ionic content of the feed solution. In the voltage–current density plots (Figure 2a), slope changes described by the I–S method can be observed for the NaCl solutions with the lower conductivities of 1, 2, and 5.5 mS/cm. Numerous plateaus were observed in the NaCl 1 mS/cm solution. The ion scarcity in this solution induced a high ED cell resistance and the absence of the Ohmic region. In contrast, the NaCl 2 mS/cm solution had a linear Ohmic region, followed by repetitive plateaus. Multiple appearances of ion-depleted regions, exhibited as numerous plateaus in both of the low concentrated NaCl solutions, indicate the hydrodynamic instabilities in the over limiting current regime [41,49]. Electroconvection is a predominant mechanism for the current transfer in dilute solutions, but with the increasing voltage the water splitting increases, suppressing electroconvection [21,39,50,51]. However, the gravitational convection has a minor effect on the LCD for the NaCl solutions of 0.02 M and lower (≤0.2 mS/cm) [50]. The NaCl 5.5 mS/cm solution did not reach the exact plateau. Nevertheless, the slope change can be clearly seen, indicating that LCD was reached (Figure 2a). The NaCl 30.6 and 48.7 mS/cm solutions remained in the Ohmic region during the whole experimental time. However, the slopes slightly increased after applying 20 and 15 V (Figure 2b) in the NaCl 30.6 and 48.7 mS/cm, respectively. This can be due to the increased ion mobility caused by Joule heat production resulting from the high current density [52]. The temperature in the NaCl 30.6 mS/cm experiment increased from 19.9 to 20.7 °C for applied 20–24.5 V, whereas in the NaCl 48.7 mS/cm it increased from 20.6 to 21 °C for applied 15–20.5 V. Of note, the highest applied voltage to solutions 4 and 5, was 24.5 and 20.5 V, respectively. At these points, the maximum allowed amperage of 5 A was reached, as a limiting current specified by the PCCell supplier.

**Figure 3:**
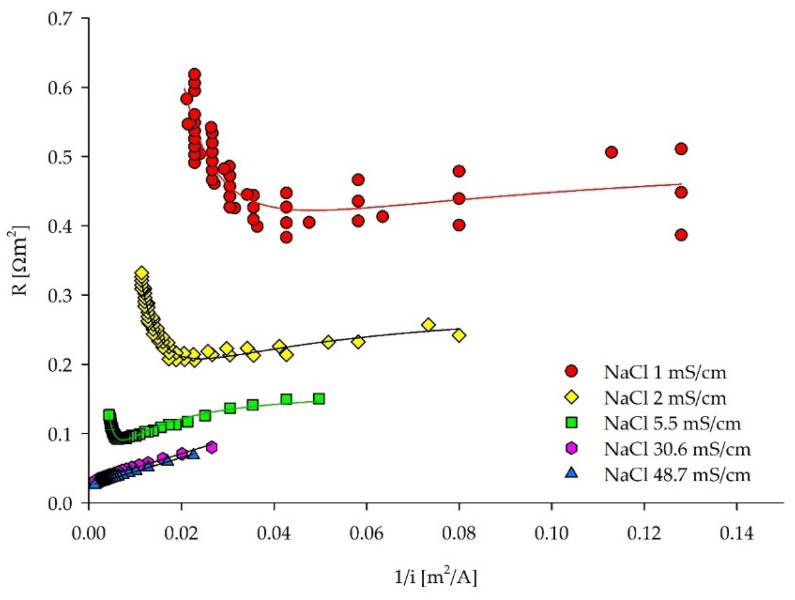
ED stack resistance–reciprocal current density curves for LCD estimation of the five NaCl solutions based on the Cowan and Brown method.

The considerable scatter in the two lower concentrated NaCl solutions can be observed in the C–B plots, similar to the I–S plots (Figure 3). Clear inflection points, indicating LCDs, for the NaCl solutions with the lower conductivities (≤5.5 mS/cm) can be deducted using the C–B approach. On the contrary, for the solutions with higher conductivities (30.6 and 48.7 mS/cm) LCD was not reached, which is in line with the results obtained from the I–S method. Experimental data were fitted by the inverse polynomial function of a second order, and LCDs were found as a function minimum for the NaCl solutions with the conductivities ≤ 5.5 mS/cm (Table 3). Fitting functions and the detailed image of the NaCl solutions of 30.6–48.7 mS/cm can be found in Supplementary Table S2 and Figure S4.

**Figure 4:**
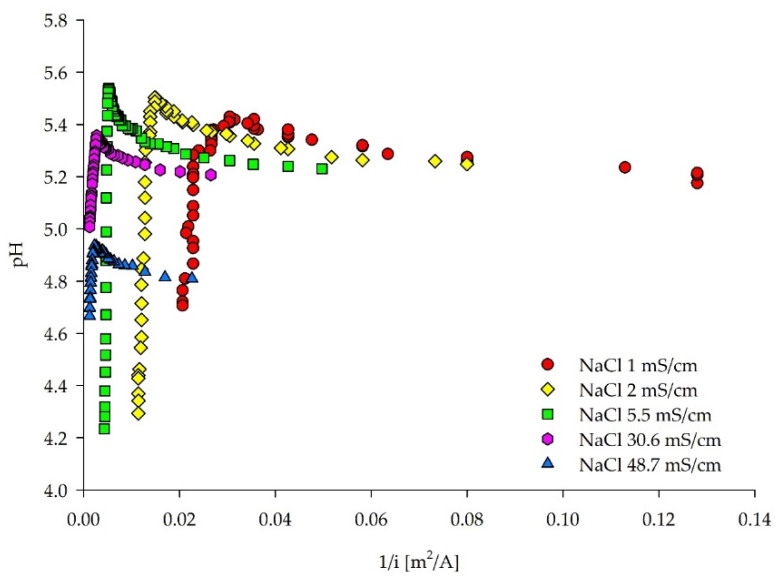
Diluate pH–reciprocal current density curves for the LCD estimation for five NaCl solutions based on the pH method. The pH changes plotted against the increasing current density can be found in Supplementary Figure S5.

Interestingly, LCDs assessed by the pH method could be successfully carried out for all of the five treated solutions, including the highest NaCl concentrations when the pH values of the same experiments were plotted (Figure 4). This behavior can be explained by the different mobilities of the Cl^−^ and Na^+^ ions [53]. Kooistra et al. [54] reported the lower LCD of a cation-exchange membrane compared to an anion-exchange membrane, as the transport number of Na^+^ is less than Cl^−^ in a NaCl solution. Moreover, the higher mobility and lower hydration number of the Cl^−^ anions would change the equilibrium state of the NaCl solution in the diluate chamber, with a sudden increase of free H^+^ ions, generating the decrease of the solution’s pH value [50]. Yet, another explanation could be found in correlation to the ED channel length. As the treated solution flows through the membrane stack, ions are removed from the diluate chamber along the whole path. With high current densities, the ions may be removed with this velocity from the frontal part of the channel, in which the solution with the depleted ions flows further through the membrane stack. Basically, at the membrane stack outlet, water dissociation occurs, induced by the low ion concentrations and high electrical potential. Research conducted on a microscale ED [42] showed concentration profiles at limiting and over limiting current densities along the length of the membrane stack. Therein, the term “vortex instability” is introduced by Kwak et al. [42]. The vortices appear in the depletion zone, and the boundary layer fluctuates with the increased current density and changes in ion concentrations along the channel. Another observation from the same research was the increase of current efficiency (λ) in the limiting regime and the occurrence of the maximum efficiency in the initial stage of the over limiting regime. Yet, La Cerva et al. [19] observed an inflection point in the λ–current density plots, with the maximum λ corresponding to the LCD.

**Figure 5:**
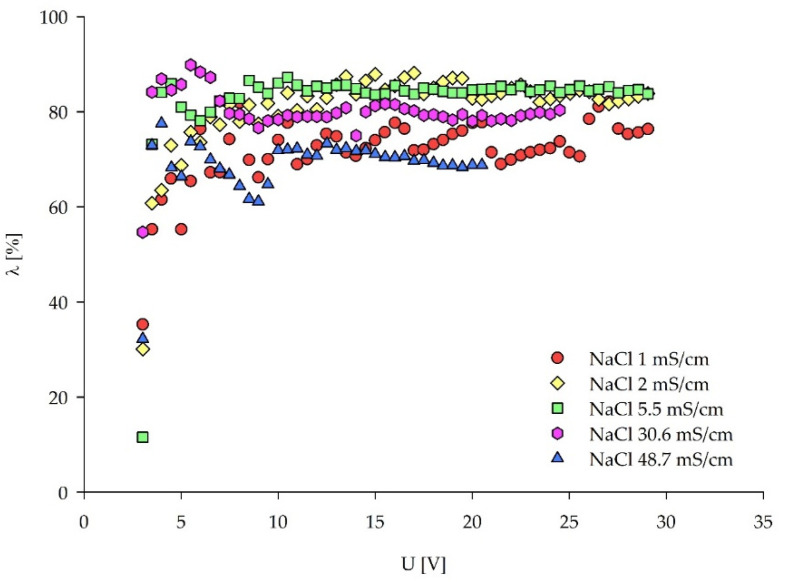
Current efficiency (λ)–voltage curves for the LCD estimation of the five NaCl solutions.

In Figure 5, current efficiency λ is plotted against the applied voltage rather than the current density to allow for a comparison between the different NaCl concentrations. However, the trend of λ values in λ–current density plot remains the same, whereas the solutions with higher NaCl concentrations develop higher current densities for the same applied voltage (Supplementary Figure S6). According to the method, the LCD can be found as the λ maximum (Table 3). However, there are no clear λ peaks indicating the over limiting current regions. This behavior deviates from the method description. Similar to the I–S plots, high λ fluctuations appear for NaCl ≤ 2 mS/cm, caused by water splitting. All of the solutions show poor λ for applied voltages lower than 3.5 V since the Faradic current is negligible beneath these voltages, similar to the results obtained by Kwak et al. [42]. For voltages above 3.5 V, all of the solutions have rather high λ, similar to the values obtained by La Cerva et al. [19] when both the concentrate and the diluate are fed with the same solutions. The highest concentrated NaCl solution manifests the lowest λ values for all of the applied voltages. This behavior is in accordance with the study of Sadrzadeh et al. [55], where λ increases with the increasing feed concentration to a maximum value and the further increase of the concentration decreases it. A sudden λ drop occurred when 5–10 V were applied to the NaCl 48.7 mS/cm solution, since the current transported by ions was not efficiently used for ionic removal from the diluate. The evolution of the current density and the ionic removal stabilized with voltages above 10 V, resulting in the stable current efficiency. Similarly, this applies to the NaCl 30.6 mS/cm solution. From the aspect of the current efficiency observed in Figure 5, optimal feed solution conductivities for the ED treatment should range from 5–30.6 mS/cm (0.03–0.3 M NaCl), for the ED set-up used in this research.

**Figure 6:**
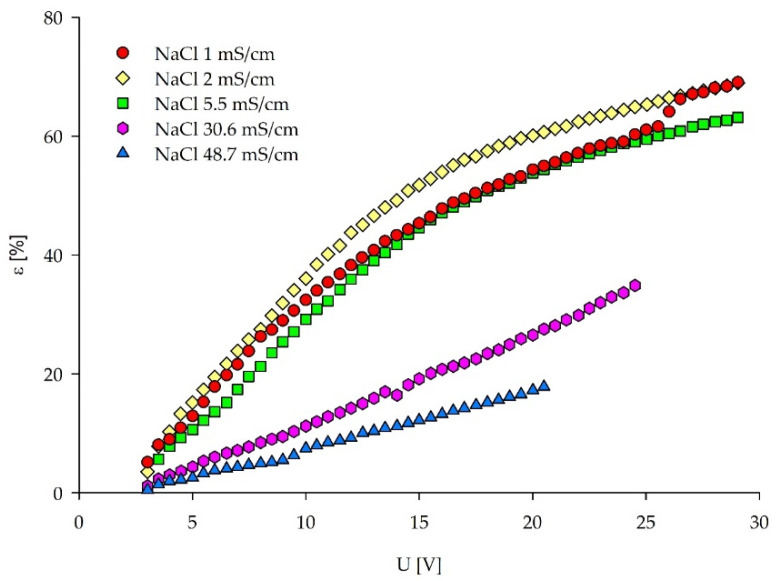
Removal efficiency (ε) for the salts present in the feed solution plotted against the applied voltage, for all of the five NaCl solutions.

LCD assessment via the removal efficiency (ε) approach (Figure 6) does not exhibit the maximum value followed by an inflection point, as described by Meng et al. [36]. However, the removal efficiency graph has similarities in this specific case with the I–S plots. Namely, a linear region (similar to the Ohmic region) can be seen for all of the feed solutions, whereas for the NaCl concentrations ≤5.5 mS/cm, a flattening of the curve introduces the second region (limiting region). Therefore, the LCDs via the removal efficiency (ε) approach can be determined as the cross-sections of these two regions. The ε values plotted against current density can be found in Supplementary Figure S7.

Values for the specific LCDs derived from all of the applied LCD assessment methods are summarized in Table 3. Temperatures vary slightly among the tested NaCl solutions. C–B LCD values are determined in three ways (Supplementary Table S3), and values denoted in Table 3 are based on the function minimum (see Section 2.2.2). The inflection point in the I–S curves was not always clearly visible in the multi-cell ED stack used in this study. The change of the overall ED stack resistance was distinctly indicated in the C–B plots. The C–B method showed the lowest LCD values compared to the other methods. Only the pH method displays LCDs for all of the five solutions if the maximum measured pH is subtracted by 0.2 pH units or the first value of the diluate pH declines. Hereby, slight increases of the diluate pH before the sudden drop can be seen in Figure 4 and Figure S3. Based on the other methods, LCD could not be determined for the NaCl ≥ 30.6 mS/cm.

### 3.2. Experiments with Different Ionic Compositions but the Same Conductivity

The graphical LCD methods were previously compared to the simple NaCl solutions to evaluate the methods and understand their coherence. The current efficiency (λ) method strongly deviated from the method description, as defined in Section 3.1. When the feed contains multiple ionic species, the λ method becomes rather impractical compared to the other graphical methods due to the required elaborate ionic analysis of the ED effluent. Consequently, this method was excluded during the subsequent analysis.

Five feed solutions (two from SSS 2.3.1, two from SCS 2.3.2, and one from RCS 2.3.3), with various ionic compositions but the same conductivity (5.5 mS/cm), were subjected to the LCD experiments and LCD assessment. The aim was to assess whether the aforementioned LCD methods apply to each type of feed representing the increasing complexity and whether the solutions with the same conductivity but different ionic compositions and complexity have the same LCD. The purpose of this approach was two-fold. The first was to evaluate the applicability of the LCD graphical methods on the more complex feed solutions, as performed for the NaCl solutions. Second, the interactions between the different ionic species as well as between the charged and uncharged molecules were expected to influence the LCD, even though the feed conductivities were the same. If this is not the case, the conductivity of the feed solution could be used as an easy-to-measure online parameter for adjusting current density during the operation under initially defined ED configurations. A 5.5 mS/cm conductivity was applied since the corresponding NaCl solution yielded LCD values by all of the used LCD graphical methods.

**Figure 7:**
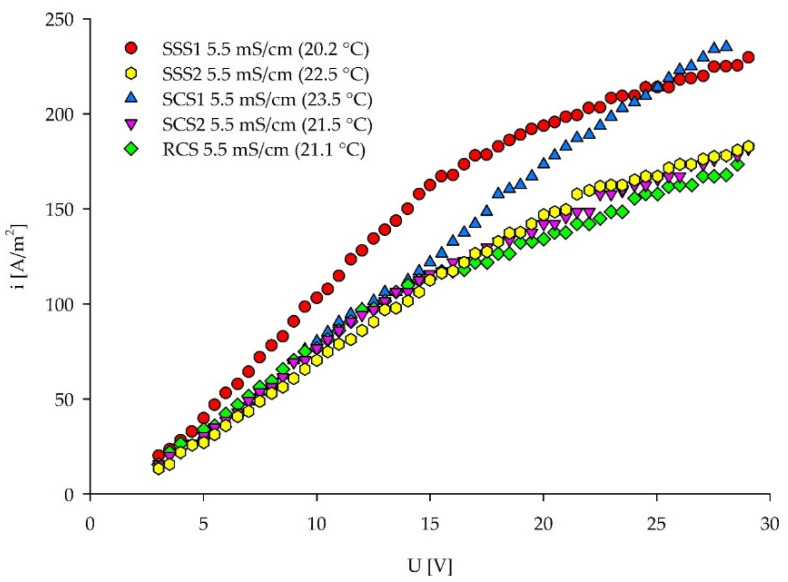
Current–voltage curves for five different solutions with the same conductivity.

An inflection point can be seen in the voltage–current density plots for all of the solutions in Figure 7, indicating the LCDs (for specific values, see Table 4). Only SCS1 does not have a clear inflection point. When the ionic composition of SCS1 and SCS2 is compared (Table 2), it can be seen that SCS1 has higher PO_4_-P and Cl^−^ concentrations, whereas SCS2 has around a double concentration of SO_4_^2−^ ions. Yan et al. [56] researched the competing transport of monovalent and multivalent ions based on their concentration ratios. In another study [57], lower LCDs were observed when some of the Cl^−^ was substituted with SO_4_^2−^ anions. Namely, sulfates have lower ionic diffusivities (0.923 × 10^−9^ m^2^/s) compared to chlorides (1.77 × 10^−9^ m^2^/s) [57]. From these studies, it can be concluded that the interactions between the ions and their molar ratio, accompanied with the elevated temperature for 2 °C, lead to the increased LCD in the SCS1 compared to the SCS2. Previous research conducted with the SCS1 solution [44] demonstrated the influence of the concentration of ions, mobility of ions, electrical potential, and the energy applied on the ion-removal rate. In those experiments, Cl^−^ showed the highest removal rate in the initial phase of the ED separation process when the higher voltage (10 V) was applied. Therefore, it can be assumed that the chloride anions were removed in the first section of the ED channel, after which other ions transport the current in the second part of the ED channel, maintaining the Ohmic region at higher voltages. Furthermore, Galama et al. [58] concluded the preferential removal of multivalent over monovalent ions at low current densities, after the demineralization rate of ~60% has been reached. Yet, another study from Benneker et al. [28] analyzed the impact of temperature gradients on the selectivity of the separation, resulting in the higher transport of divalent Mg^2+^ than the monovalent K^+^ and Na^+^ ions with an elevated solution temperature. This behavior may impact the ionic transport since the temperature inside the ED stack increases with the increasing current density. However, they assessed the temperature gradient of ±20 °C, whereas in our experiments it does not exceed ±2 °C, and thus can be neglected.

**Figure 8:**
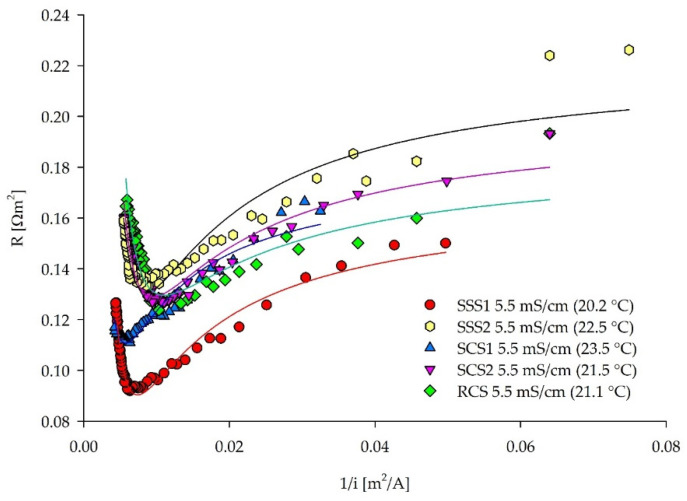
ED stack resistance–reciprocal current density curves for the LCD assessment of five different solutions with the same conductivity.

For all of the solutions, an exact inflection point can be identified by applying the C–B approach (Figure 8). With the increasing solution resistance, LCD is generally decreasing (Table 4). The SSS1 (NaCl) solution has the lowest resistance, showing the higher mobility of the Na^+^ and Cl^−^ ions, compared to the SSS2 (Na_2_SO_4_) solution and complex media (SCS1–2) with the same measured conductivity. The SSS2 solution has the highest resistance, followed by the real fermentation wastewater sample (RCS), with the same conductivity of 5.5 mS/cm. These high resistances can be explained in the first order by the presence of SO_4_^2−^ ions. The dominant presence of SO_4_^2−^ ions was the main reason for the higher solution resistance and lower LCDs, as can be seen in Figure 7 and Figure 8, especially comparing SSS1 (NaCl) and SSS2 (Na_2_SO_4_) solution. When SCS2 and RCS are compared, other non-charged substances (e.g., organic carbon) originating from culture media and microbial excretions seem not to affect the overall resistance. Different ionic species in various quantities significantly impact on the solution’s resistance in the membrane stack.

**Figure 9:**
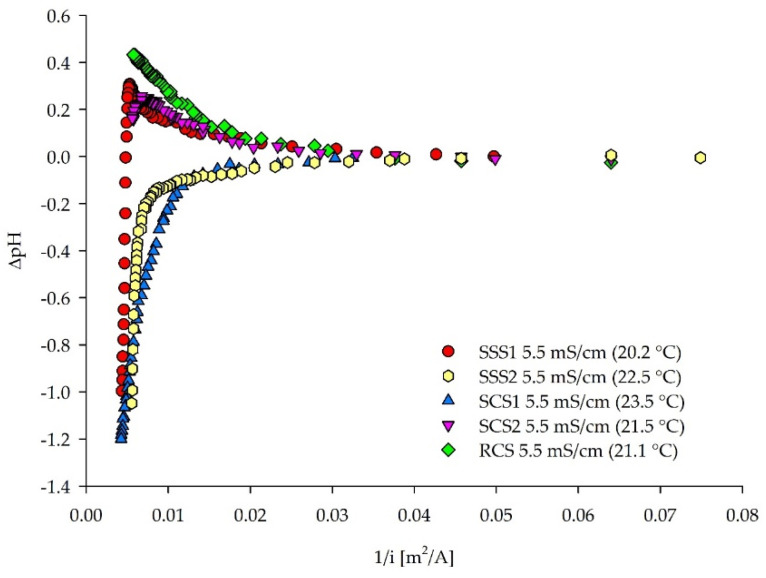
The ΔpH values plotted against the reciprocal current density for five different feed solutions.

A *ΔpH* axis (see Section 2.2.3) was applied for the LCD assessment by the pH method (Figure 9), since the initial pH values of compared solutions vary strongly between each other (pH 2.2–8.5). Culture media for fermentation usually include buffering compounds, such as phosphates for pH stability [59]. The gradual diluate pH drop of the SCS1 (Figure 9) can be explained by its dominant PO_4_^3−^ concentrations (Table 2), acting as a buffer, accepting that H^+^ ions evolved due to the “water splitting” effect. Similarly, ammonium can buffer OH^−^ ions that are evolved on the CEM-side by donating a proton to form water molecules. On the contrary, the pH drop is rather sudden in the case of single salt solutions (SSS1–2). An increase of the diluate pH can be seen with the increasing current density (voltage) when comparing the model (SCS2) and real fermentation wastewater (RSC). The initial pH increase in the SCS2 was followed by a sudden pH drop induced at 112.5 A/m^2^, which is not the case for the RSC. This indicates the effect of the other compounds present in the real samples on the solution characteristics and behavior during the ED treatment, resulting in a continuous pH increase. Organic nitrogen can also provide a buffering capacity, such as proteins [60], present in the RCS but not in the SCS2, preventing steep pH changes. The LCD could be defined only by a modified pH method requirement, with ±0.2 pH units change in the diluate. Therefore, Table 4 (pH method) shows that the LCD decreases with the increase of the solution complexity.

**Figure 10:**
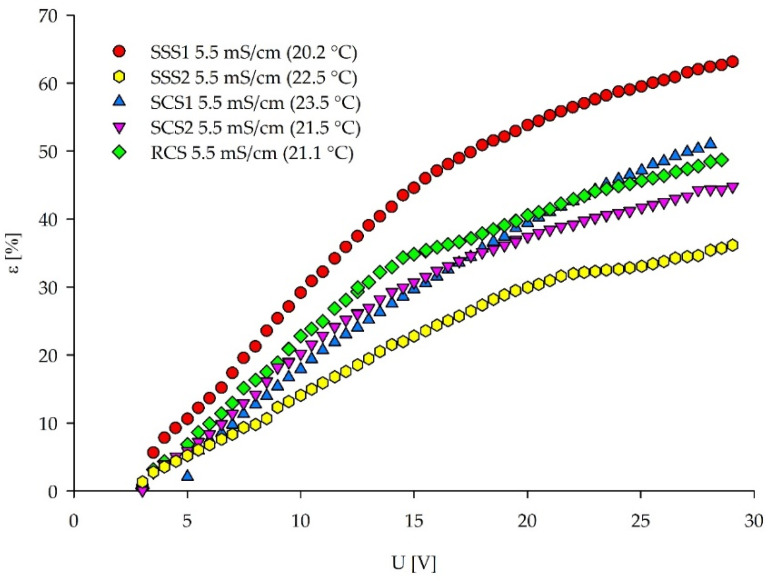
Removal efficiency for the salts present in the feed plotted against the applied voltage.

With the increasing applied voltage, the salt removal efficiency (Figure 10) can be divided into two stages according to the I–S current–voltage curves, with a linear region (Ohmic dependency) transfiguring into the limiting region (flattening of the curve). LCD was detected at the intersection of these two stages (Table 4). SSS1 (NaCl) achieved the highest removal efficiencies, whereas SSS2 had the lowest. The presence of the SO_4_^2−^ is impeding the removal efficiency, due to the higher overall resistance of the ED stack and prevailing concentration polarization effect at the membrane surface, although the initially measured conductivities were the same for all of the tested solutions. The removal efficiencies (ε) of complex solutions are in the same range, corresponding to their similar content of equivalent multivalent anions (Table 2). The ε values plotted against current density can be found in Supplementary Figure S8. In these plots, the maximum ε was not followed by an inflection point, which would indicate the occurrence of the LCD.

Table 4 summarizes the final LCD values for the solutions derived from the graphical assessment (Figure 7, Figure 8, Figure 9 and Figure 10) of the previous diagrams. The removal efficiency method (ε) had a similar trend to the I–S method, facing the same LCD estimation problems of distinguishing between the Ohmic and limiting regions. On the one hand, the pH changes were an indicator for different removal velocities of anionic and cationic species. On the other hand, the changes indicated “water splitting” and unfavorable H^+^ production. However, the pH changes depend on the initial solution pH and composition. Therefore, the pH method should be used as a secondary LCD measurement. The C–B method expressed the most reliable and unique values, as the sudden increase of the ED stack resistance was clearly visible for all of the solutions with different complexities. This inflection point can be correlated with the boundary layer formation on the diluate side. The standard error of experimental data analyzed by the C–B method was less than 0.03, providing good quality LCD assessment fitted with the inverse second-order polynomial function (Supplementary). The I–S method yielded higher LCD values, and a clear inflection point was not always visible. Therefore, estimation of the LCD was not unambiguous. The quality of the LCD assessment by the I–S method depends on the length of the data points selected for the estimation of the intersecting point and the intersection of two linear functions. As a result, the error of the data analysis can be higher compared to the C–B method. Finally, the detection of the LCD was more pronounced in the C–B method than in the I–S method. The LCD values assessed via the C–B method were used to compare the conductivity–composition–LCD relations among the analyzed solutions. The SSS2 (Na_2_SO_4_) solution had lower LCD (119.3 A/m^2^) compared to the SSS1 (NaCl) solution (130.9 A/m^2^), even though the conductivities were the same. Due to the larger SO_4_^2−^ size, and their lower mobility, compared to the Cl^−^, the overall resistance was higher, and the boundary layer formed earlier. The SCS1 solution had a higher LCD than SSS1 according to Table 4, although the solution comprised various ions and had higher overall resistance. This was not the case when the LCDs tracked as the resistance minimum were compared (Supplementary Table S4), with 159.7 and 157.8 A/m^2^ for SSS1 and SCS2, respectively. Moreover, one smaller inflection point of SCS1 can be seen at 0.011 m^2^/A (90.5 A/m^2^) (Figure 8). Since both the model and real fermentation wastewater had a high SO_4_^2−^ concentration (Table 2), the lower LCDs correspond to the lower LCD of the SSS2, as denoted above. The negatively charged organic molecules, most probably present in the real wastewater [1], may also impact the reduction of the LCD by blocking the way of the anionic migration through the anion-exchange membranes. More in-depth research on a molecular level is necessary to understand the behavior of complex feed solutions in the limiting and over limiting regions. Nevertheless, this work focuses on the general approach for LCD determination and the solutions assessed here had the same trend in the C–B plots.

### 3.3. Same Ionic Composition but Different Initial Conductivity

**Figure 11:**
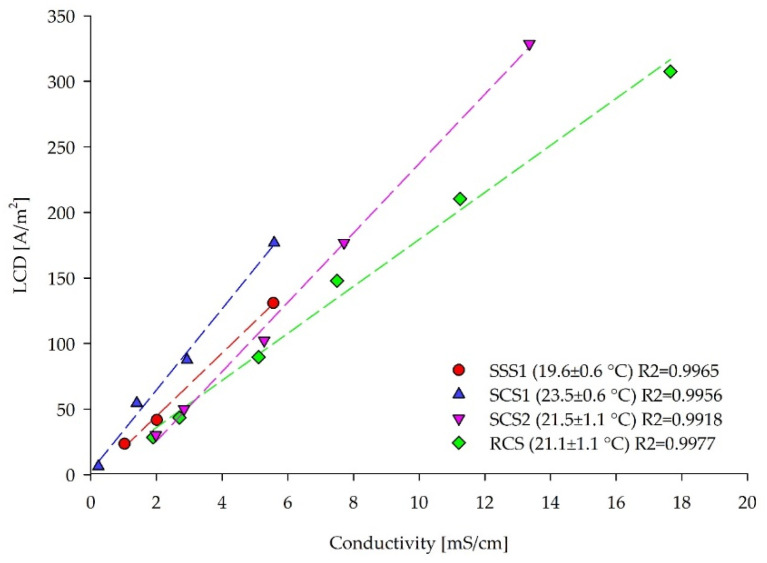
Dependency of the inlet feed conductivities (mS/cm) of the dilutions from different solutions and corresponding LCDs assessed by the Cowan and Brown method. During the batch ED, the feed concentration continuously decreases, consequently, the LCD decreases.

Due to the unambiguity of the results obtained in Section 3.1 and Section 3.2, the C–B method was applied to a span of feed solutions (SSS, SCS, RCS) with increasing–decreasing salt concentrations. The dilution ranges for the LCD experiments were prepared to mimic the decreasing ionic content of the diluate in the batch ED. The LCD values were successfully obtained by the C–B method for all of the tested solutions with the inlet feed conductivities ≤18 mS/cm (Figure 11). LCD correlates linearly to the feed conductivity, with a high fitting accuracy of R^2^ = 0.99 for SSS1, SCS1, SCS2, and RCS, similar to the literature values [16,32,53].

As shown in Figure 11, the LCD–conductivity correlation for RCS has the smallest slope, compared to the model solutions containing only salts (SSS, SCS). The SCS2 solution had 1.3 times higher LCDs for every increase of 1 mS/cm of measured inlet conductivity compared to the RCS, whereas for SSS1 and SCS1 this ratio was 1.2 and 1.7, respectively. This behavior clearly indicates the retarding effect of other compounds present in real waste streams, such as organic molecules, on the ionic removal from the feed solution. Therefore, the LCD decreased, even though the ionic strength was the same. The current density applied to the ED membrane stack could be gradually reduced with the decreasing diluate concentration in the batch ED process for one type of feed solution and set-up. However, ion size and mobility, ion concentration, concentration ratios of the different ions, and organic components of the treated feed impact the LCD and ion removal ratio. Therefore, conductivity alone cannot be used for the LCD determination among various feed solutions.

## 4. Summary

This work focused on comparing five graphical methods for the limiting current density (LCD) assessment in the batch electrodialysis desalination process (ED). The applied methods were: Isaacson and Sonin (current density–voltage), Cowan and Brown (ED stack resistance–reciprocal current density), pH method (diluate pH–reciprocal current density), λ method (current efficiency–voltage), ε method (removal efficiency–voltage).

First, five methods were applied to five different NaCl concentrations, representing the solutions with the lowest level of complexity. The λ method did not exhibit strict LCD values, as described by the method. The pH method revealed LCDs for all of the five concentration levels, indicating “water splitting” occurrence. The other three methods demonstrated LCD only for the lower NaCl concentrations (≤0.03 M NaCl), whereas for the concentrations ≥0.3 M NaCl limiting values could not be obtained, as the ED equipment has the maximum tolerating amperage of 5 A.

Second, the applicability of four LCD methods (λ method was excluded from this part) was tested in solutions with the same conductivity but different levels of complexity (synthetically prepared and real waste streams). Due to the presence of different ionic species, comprising different sizes and mobilities, the resistances of the ED stack differed, although the measured conductivities were the same. Therefore, the obtained LCDs varied, as well. The presence of the SO_4_^2−^ anions may be lowering the solution’s LCD. However, in-depth research on the molecular level is necessary, to understand the behavior of complex feed solutions in the limiting and over limiting regions. Other compounds, such as large organic molecules in the real waste streams, decrease the LCD. The pH method applied to the fermentation wastewater did not reveal a sudden pH drop of the diluate with the increased voltages, deviating from the typical behavior of the NaCl solution. Other methods worked in accordance with the assessments conducted on the simple NaCl solution.

Third, a linear dependence of the LCD determined by the Cowan and Brown method and the measured solution conductivity was concluded for various dilution steps of each solution type. However, the gradual decrease of the current density applied to the ED stack cannot be conducted solely by monitoring the diluate conductivity, since the LCD depends on the present ionic species and other compounds, such as organic molecules in the real waste stream.

## 5. Conclusions

Based on the initially presented research questions, the following conclusions can be drawn from this research:

- All of the assessed graphical methods showed LCDs for the feed concentrations ≤0.03 M NaCl. The current efficiency (λ) method did not have a clear peak at the LCD as defined by the method. Only the pH method revealed LCDs for feed concentrations ≥0.3 M NaCl.
- Four out of five LCD methods were applicable to each type of feed with increasing complexity. The current efficiency (λ) method was excluded, and the pH method was modified (±0.2 pH units from the initial feed pH). The C–B method had the most consistent results for all of the different types of feed solutions, and lower LCD values compared to the I–S method. In conclusion, the usage of the C–B method is highly recommended due to its applicability to a wide range of solutions with varying ionic compositions, concentrations, and matrix complexities.
- Solutions with the same conductivity but different ionic compositions do not reach the same LCD, as it is strongly influenced by the type and concentration of ionic species, and the presence of uncharged compounds.
- In one solution matrix, LCD linearly correlates with the decreasing conductivity. Therefore, the online conductivity measurement could be used for the batch ED control of one specific medium with a known composition. Otherwise, measuring only the conductivity is not suitable for current density adjustment unless combined with other characteristics of the feed solution.
- The obtained results bring knowledge to the existing graphical LCD methods and the complex medias’ behavior during electrodialysis membrane separation processes. The conclusions obtained in this work are useful for the selection of an appropriate LCD method in real feed solutions, LCD assessment, and future automatization of the electrodialysis.

## Figures and Tables

**Table 1:** Characteristics of the ion-exchange membranes, as specified by the PCCell producer.

Membrane	Type	Thickness, µm	Transference Number	Resistance, Ohm cm^2^	Water Content (wt%)	pH Stability
PC SA	Anion-exchange	100–110	>0.95	1.8	14	0–9
PC SK	Cation-exchange	100–120	>0.95	2.5	9	0–11
PC MTE	End membrane	220	>0.94	4.5	-	1–13

**Table 2:** Total organic carbon (TOC) and ionic content of the initial synthetic and real solutions with corresponding conductivity.

Solution	TOC	PO_4_-P	Cl^−^	SO_4_^2−^	NH_4_-N	Na^+^	K^+^	Ca^2+^	Mg^2+^	Conductivity
	mg/L	mmol/L								mS/cm
SSS1	0	0	53.6	0	0	53.8	0	0	0	5.5
SSS2	0	0	0	19.5	0	9.4	0	0	0	5.5
SCS1	0	13.2	12.0	12.1	12.8	11.9	13.2	0	2.8	5.5
SCS2	0	4.3	2.01	71.0	92.2	25.7	2.8	0.8	1.0	13.8
RCS	1060	4.1	2.2	85.1	94.4	37.5	4.7	1.3	2.5	18.4

**Table 3:** List of solutions (SSS—2.3.1) used in the study with corresponding LCDs defined by five methods.

Solution	NaCl 1	NaCl 2	NaCl 5.5	NaCl 30.6	NaCl 48.7
**NaCl concentration [mol/L]**	0.005	0.004	0.03	0.3	0.5
**Conductivity [mS/cm]**	1	2	5.5	30.6	48.7
**Average temperature T [°C]**	19.1	19.2	20.2	20.4	21.0
**Initial pH**	5.16	5.23	5.21	5.20	4.80
**LCDs [A/m^2^]**					
**I–S**	30.1	58.8	175.5	/	/
**C–B**	23.5	41.7	130.9	/	/
**pH Method**	43.8	78.1	209.6	771.2	/
Max pH *	43.8	72.9	203.1	554.7	693.6
pH decrease **	34.2	67.2	198.4	382.8	459.7
λ Method ***	43.7	67.6	107.8	108.0	78.1
**ε Method**	23.4	42.8	167.5	/	/

* LCD values as the maximum measured pH value subtracted by 0.2 pH units. ** LCD values recorded for the first diluate pH decline. *** LCD values as the maximum λ values.

**Table 4:** List of solutions used in the study (Section 2.3.1, Section 2.3.2 and Section 2.3.3) with corresponding LCDs defined by four methods.

Solution	SSS1 (NaCl)	SSS2 (Na_2_SO_4_)	SCS1	SCS2	RCS
**Conductivity [mS/cm]**	5.5	5.5	5.5	5.5	5.5
**Average temperature [°C]**	20.2	22.5	23.5	21.5	21.1
**Initial pH**	6.31	8.49	4.59	3.24	2.25
**LCD [A/m^2^]**					
**I&S**	175.5	153.2	179.0	135.7	117.2
**C&B**	130.9	119.3	176.8	102.3	89.6
**pH method**	209.6	137.6	90.5	(112.5) *	(76.5) *
**ε Method**	167.5	158.3	137.4	118.0	112.9

* LCD values corresponding to the increase of the measured pH by 0.2 pH units from the initial value.

## Supplementary Materials

The following supporting information can be downloaded at: https://www.mdpi.com/article/10.3390/membranes12020241/s1, Figure S1: 10-cell pair lab-scale ED unit (PCCell ED 64-004, Heusweiler, Germany); Figure S2: NaCl concentration-conductivity diagram; Figure S3: The intersection of two fitting linear functions for the LCD determination in (a) NaCl 1 mS/cm solution; (b) NaCl 2 mS/cm solution; (c) NaCl 5.5 mS/cm solution; Figure S4: ED stack resistance–reciprocal current density curves for LCD of two high concentrated NaCl solutions based on the Cowan and Brown method. With the increasing current density, the resistance is decreasing and the inflection point does not appear for these two solutions; Figure S5: Change of the diluate pH with (a) current density and (b) applied voltage; Figure S6: Current efficiency (λ)–current density curves for LCD estimation of five NaCl solutions; Figure S7: Removal efficiency (ε) for the salts present in the feed solution plotted against current density, for all five NaCl solutions; Figure S8: Removal efficiency for the salts present in the feed plotted against current density. Table S1: The slopes (m), intercepts (b) and the coefficient of determination (R2) for two fitting linear equations in experimental data of three NaCl solutions for the LCD determination; Table S2: Coefficients of the inverse polynomial function of a second order for estimating LCDs based on Cowan and Brown method presented in the research paper; Table S3: List of solutions (SSS–2.3.1) used in the study with corresponding LCDs defined by Cowan and Brown method in three ways (a/b/c); Table S4: List of solutions used in the study (Chapters 2.3.1., 2.3.2., 2.3.3.) with corresponding LCDs defined by Cowan and Brown method in three ways (a/b/c).

## Appendix A

This section describes the graphical LCD methods used to determine LCDs in various solutions and compares their outcomes to find the most reliable LCD method for the real feed streams.

### Appendix A.1. Method by Isaacson and Sonin

The Isaacson and Sonin (I–S) method analyzes the current density (mA/cm^2^) behavior depending on the voltage applied to the ED cell. Three different regions (I–III) are defined by this method (Figure A1). (I) is the Ohmic region with a linear correlation of the current density with the applied voltage. (II) is a region where the linear dependence starts leveling out. Here, the operational mode is transfiguring to the limiting current density region, indicating an energy loss due to the increased voltage resulting in the splitting of water molecules to H+ and OH- ions, gravitational convection, and electroconvection. The intersection point of the two slopes belonging to the Ohmic and the second (plateau) region indicates the LCD. The third region (III) extends over the increasing positive slope, where a second intersection point can be determined. This region can be explained by the contribution of already split water molecules to the conducting electricity through the ED cell, gravitational convection, and electroconvection.

### Appendix A.2. Method by Cowan and Brown

In the LCD method by Cowan and Brown (C–B), the ED cell resistance from the experimental data is plotted against the reciprocal current density (Figure A2). LCD manifests at a polarization voltage as a rapid change of the slope. The ED cell resistance decreases with the increase of the current density up to a certain point. At this point, with further increase of the current density (applied voltage), regions with higher resistance in the ED cell evolve, drawing a negative slope on the diagram. The intersection of the positive and the negative slope represents the LCD.

### Appendix A.3. The pH Method

It was first noticed by Cowan and Brown (Cowan and Brown 1959) that the pH of the diluting stream starts to decrease at a current value very close to the value at which the resistance slope changes. Therefore, the authors (Melin and Rautenbach 2007) define the limiting current density for the pH method as a point where the pH value of the diluting stream shows a decrease of 0.2 pH units. This breaking point can be seen on the diluate pH–reciprocal current density diagram (Figure A3).

### Appendix A.4. Current Efficiency Method

La Cerva et al. (La Cerva et al. 2018) examined the research of Kwak et al. (Kwak et al. 2013) and suggested plotting the current efficiency (*λ*) against the current density, wherein the LCD appears at the maximum *λ* (%) (Figure A4). The *λ* was calculated according to the following equation:

(A1) $$\lambda =\frac{zFQ_{dil}\left(C_{dil}^{IN}-C_{dil}^{OUT}\right)}{NI}\;\cdot 100\;\%$$

where *z* is the ionic charge, *F* is Faraday constant (96485.33 sA/mol), *Q_dil_* is the flow of the diluate stream (L/s), *C_dil_^IN^* and *C_dil_^OUT^* are the inlet and outlet concentrations of the diluate stream (mol/L), *I* is the current value (A), and *N* is the number of the ED cell pairs.

### Appendix A.5. Desalting Efficiency Method

Meng et al. (Meng et al. 2005) proposed a method for the determination of the optimal operating current based on the desalting efficiency (*ε*):

(A2) $$\varepsilon =\frac{\kappa_{dil}^{IN}-\kappa_{dil}^{OUT}}{\kappa_{dil}^{IN}}\;\cdot 100\;\%$$

where *κ_dil_^IN^* is the initial conductivity (mS/cm) of the feed to be treated, and *κ_dil_^OUT^* is the conductivity (mS/cm) of diluate generated by the ED. Values of the desalting efficiency are plotted against the current density, wherein the LCD appears as the maximum *ε* (%), representing the optimal operating current (Figure A5).
