# Supplementary material for: Assessment of Graphical Methods for Determination of the Limiting Current Density in Complex Electrodialysis-Feed Solutions
Source: Membranes (Basel). 2022 Feb 18;12(2):241. doi: 10.3390/membranes12020241 (PMC8875246; doi:10.3390/membranes12020241)
Supplement: Supplementary file 1 [file membranes-12-00241-s001.zip › membranes-1580097-supplementary.pdf]

Supplementary

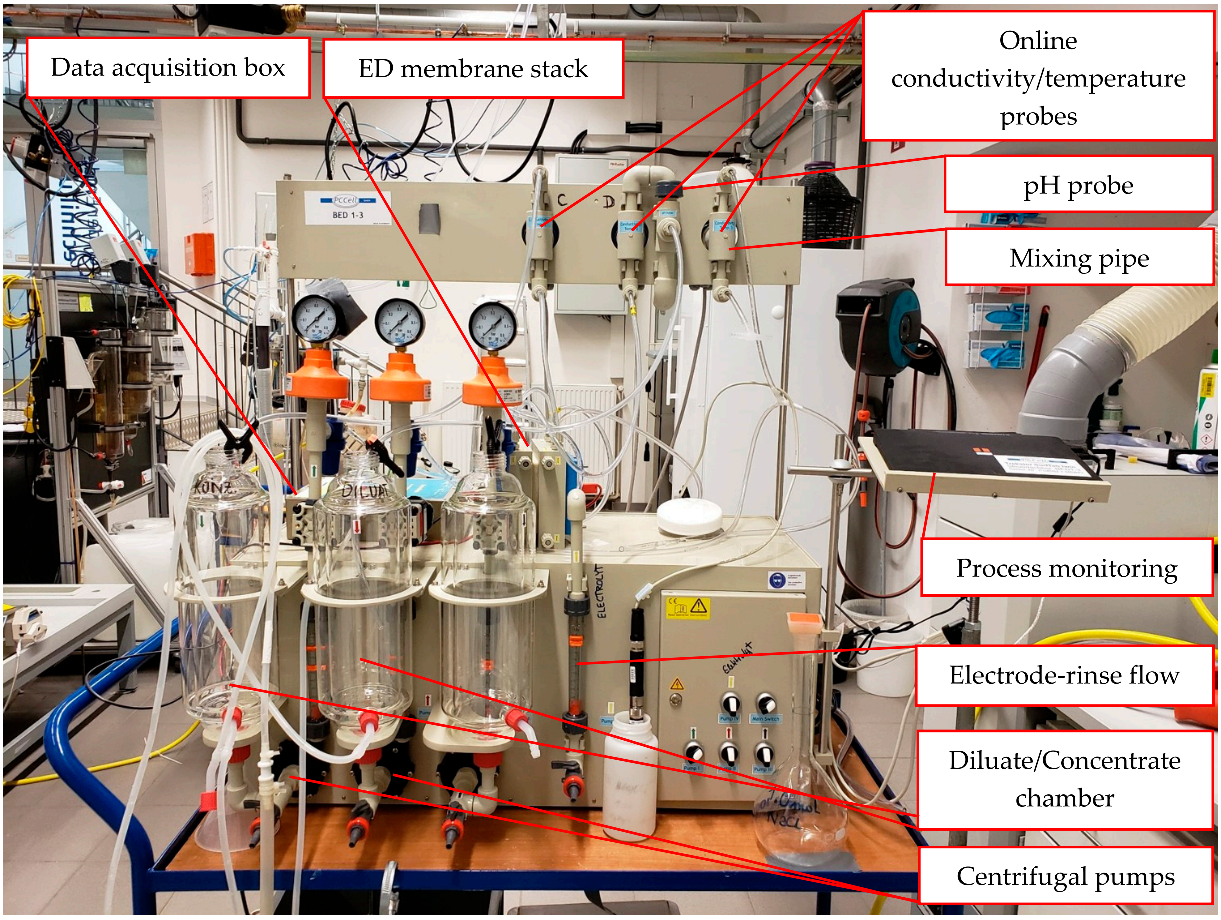

Figure S1. 10-cell pair lab-scale ED unit (PCCell ED 64-004, Heusweiler, Germany)

The resistance of an ED stack depends on the number and type of the ion-exchange membranes (cation, anion and end membranes), the specific membrane resistance and active membrane area, the type and ionic concentration of the feed solution, spacers and the resistance of the electrode-rinsing solution and electrodes. The membrane active area in the used ED stack was 64 cm^2, and for the nine cation-exchange membranes, ten anion-exchange membranes and two end membranes the total resistance was 0.8 Ω. The specifications of membranes are in the main document in Table 1. The resistance of the 0.25 M Na_2SO_4 electrode-rinse solution is estimated to be 4.2 Ω. These values are taken as constant values for the used ED stack. The resistance of the electrodes and spacers is often neglected in the calculations of the total ED stack resistance. Further on, the resistance of the diluate and concentrate chamber are calculated from the online conductivity measurements as follows:

$$R = \frac{1}{\kappa} \cdot \frac{h}{A_{eff}} \quad \text{(S1)}$$

Where the κ (mS/cm) is the specific conductivity of a solution in either diluate or concentrate chamber; h (cm) is the distance between the membranes, and A_{eff} (cm^2) is the active membrane area. From Equation S1 can be seen that the resistance of the treated solution changes during the ED demineralization time, increasing in the diluate chamber and decreasing in the concentrate chamber. Also, the resistances of the solutions employed in this study differed strongly among NaCl solutions with the conductivities of 1–48.7 mS/cm. The contribution of the solution resistance varied from 26–6% of the total ED stack resistance for the NaCl solutions of 1–48.7 mS/cm, respectively. The total ED stack resistance can be clearly seen in the Cowan & Brown plots. Therefore, the relative contribution of the membranes' resistance to the total ED stack's resistance increased averagely from 1–15% with increasing salt concentration represented by the increasing conductivities of 1–48.7 mS/cm, respectively. For example, the contribution of the membrane resistance was 4.6% and of the solution resistance was 20.4% in the NaCl 5.5 mS/cm solution. The formation of the boundary layer can be seen at the resistance inflection point in the Cowan & Brown plots for the NaCl solutions with the conductivity ≤ 5.5 mS/cm. The contribution of the boundary layer resistance to the total ED stack resistance increased with the further voltage increase.

For the preparation of the NaCl dilution steps measured conductivities and NaCl concentrations were recorded, having a linear dependency (Figure S2).

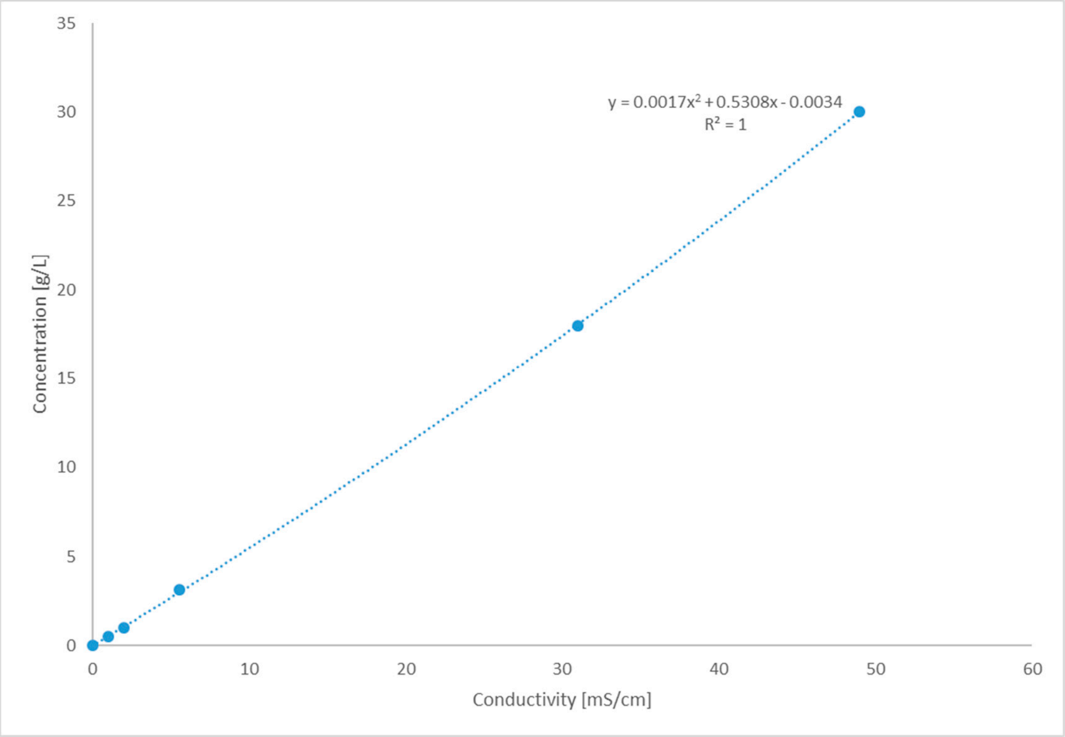

Figure S2. NaCl concentration - conductivity diagram

In Figure S2 are the experimental data of the NaCl solutions with the conductivities ≤ 5.5 mS/cm. The data were fitted to linear functions to find the LCD based on the I&S method.

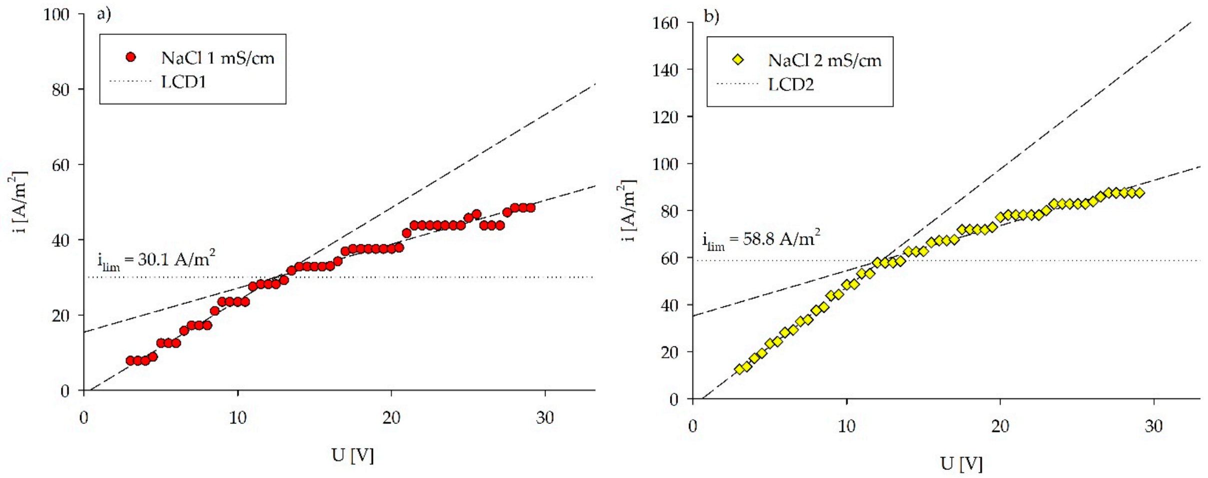

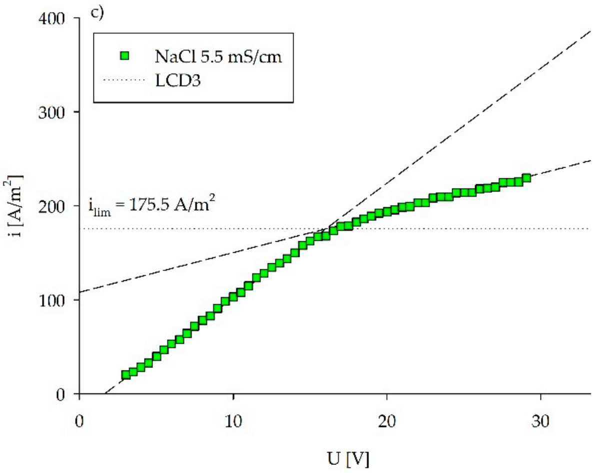

Figure S3. The intersection of two fitting linear functions for the LCD determination in a) NaCl 1 mS/cm solution; b) NaCl 2 mS/cm solution; c) NaCl 5.5 mS/cm solution.

In Table S1 are the parameters of linear functions f(x) = mx + b that were used for fitting the experimental data points in the NaCl solutions with the conductivities ≤ 5.5 mS/cm.

Table S1. The slopes (m), intercepts (b) and the coefficient of determination (R2) for two fitting linear equations in experimental data of three NaCl solutions for the LCD determination.

Solution	m_1	b_1	R_1^2	m_2	b_2	R_2^2
NaCl 1 mS/cm	2.4744	-0.9438	0.9619	1.1678	15.4490	0.9455
NaCl 2 mS/cm	5.0314	-2.9481	0.9947	1.9238	35.2072	0.9670
NaCl 5.5 mS/cm	12.2057	-19.7312	0.9990	4.2176	108.0440	0.981

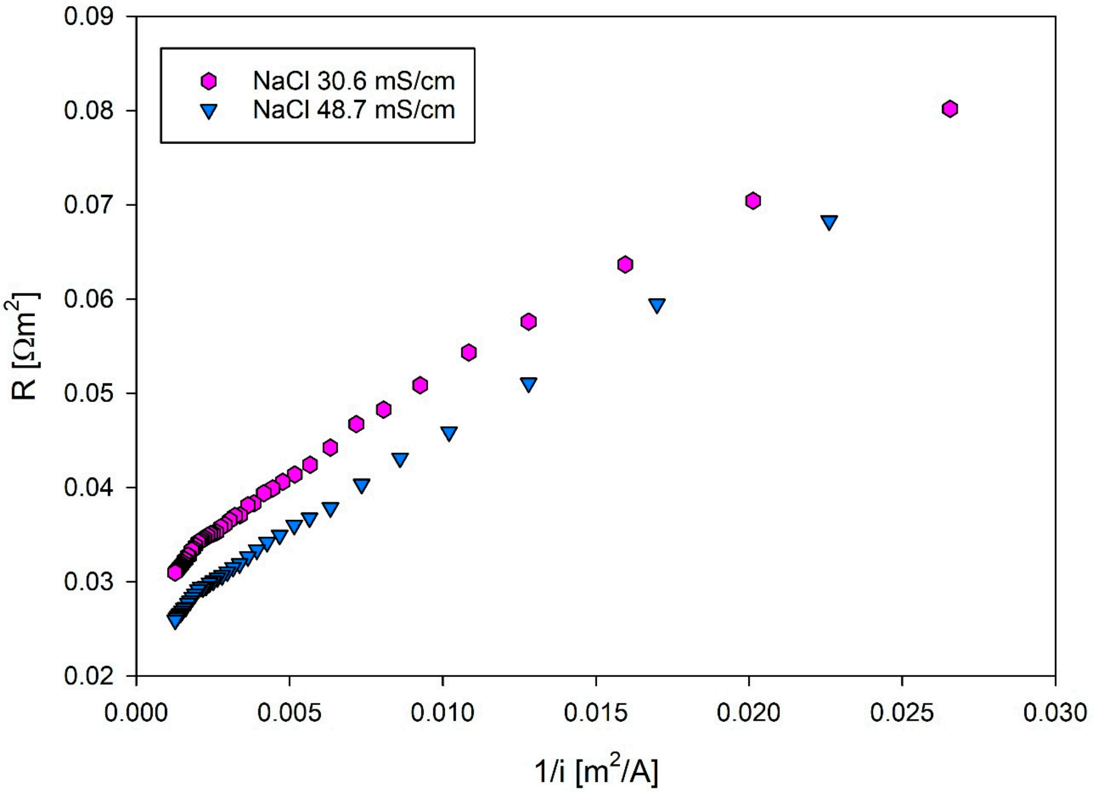

Figure S4. ED stack resistance – reciprocal current density curves for LCD of two high concentrated NaCl solutions based on the Cowan and Brown method. With the increasing current density, the resistance is decreasing and the inflection point does not appear for these two solutions

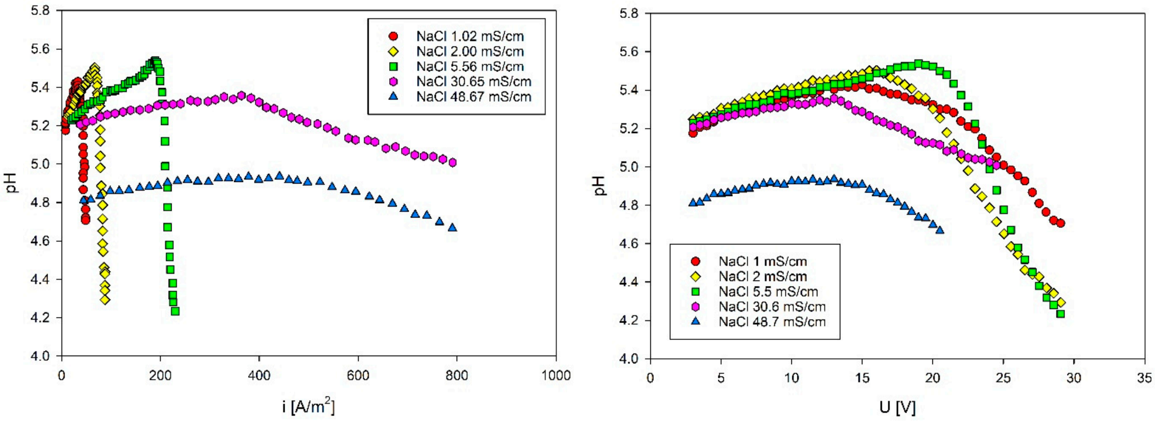

a) b)

Figure S5. Change of the diluate pH with a) current density and b) applied voltage

The more intuitive approach of LCD assessment based on the pH value is shown in Figure S4, where the diluate pH is correlated either to the applied voltage (Figure S4a) or to the current density (Figure S4b). A clear drop in the diluate pH can be seen for all five treated NaCl solutions. The corresponding LCD values are in the Table 2, estimated in three ways. The LCD values adopted for the first diluate pH decline have the values between pH Method (drop for 0.2 pH units) and Max pH (max pH value subtracted for 0.2 pH units). Still, all LCDs indicate higher values compared to the C&B method.

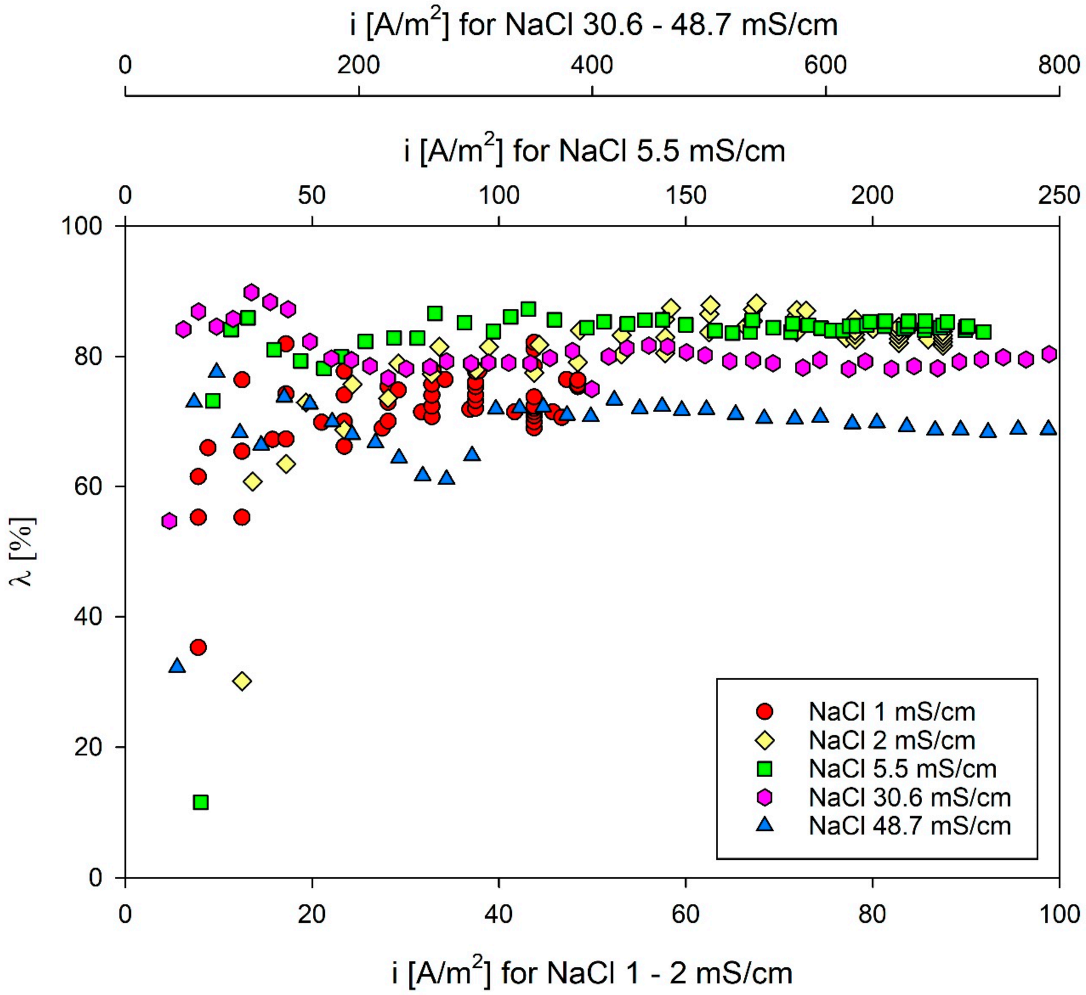

Figure S6. Current efficiency (λ) – current density curves for LCD estimation of five NaCl solutions

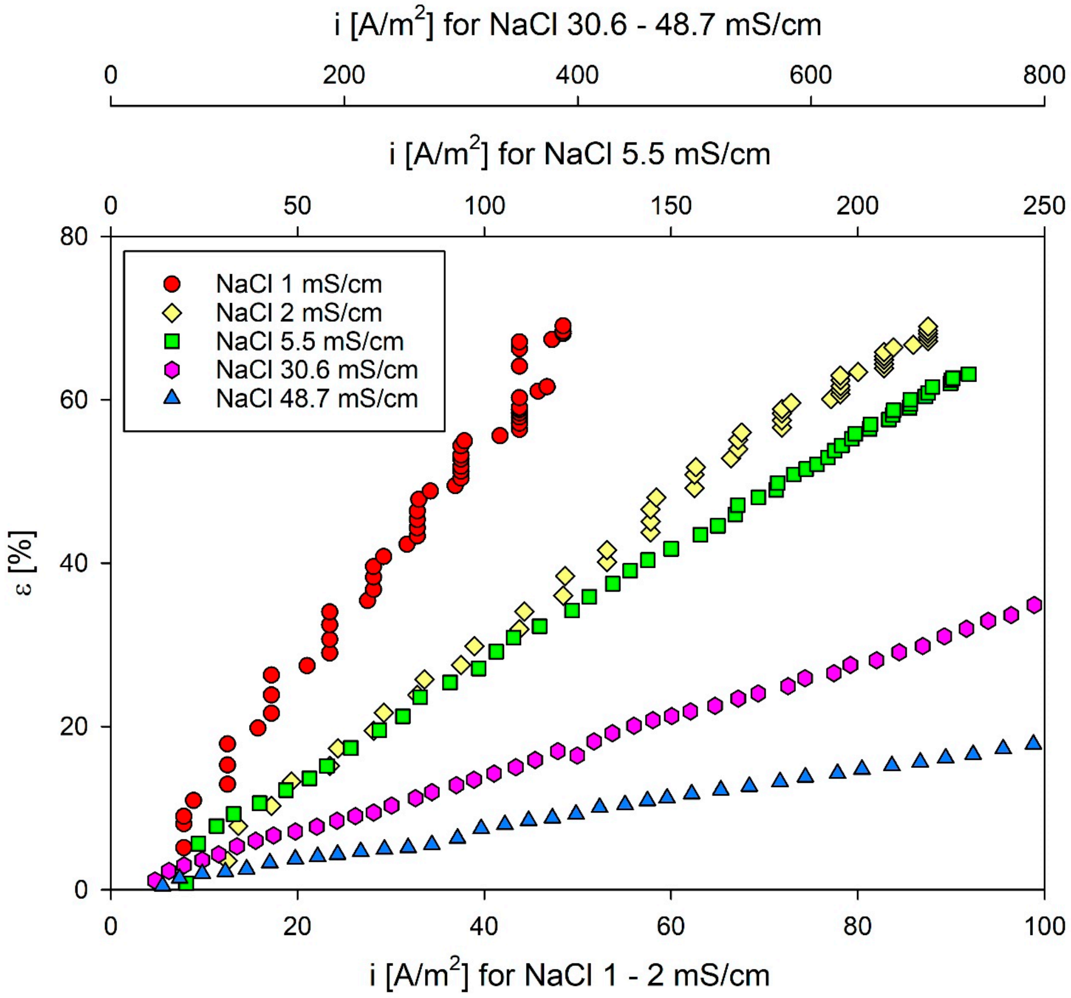

Figure S7. Removal efficiency (ε) for the salts present in the feed solution plotted against current density, for all five NaCl solutions.

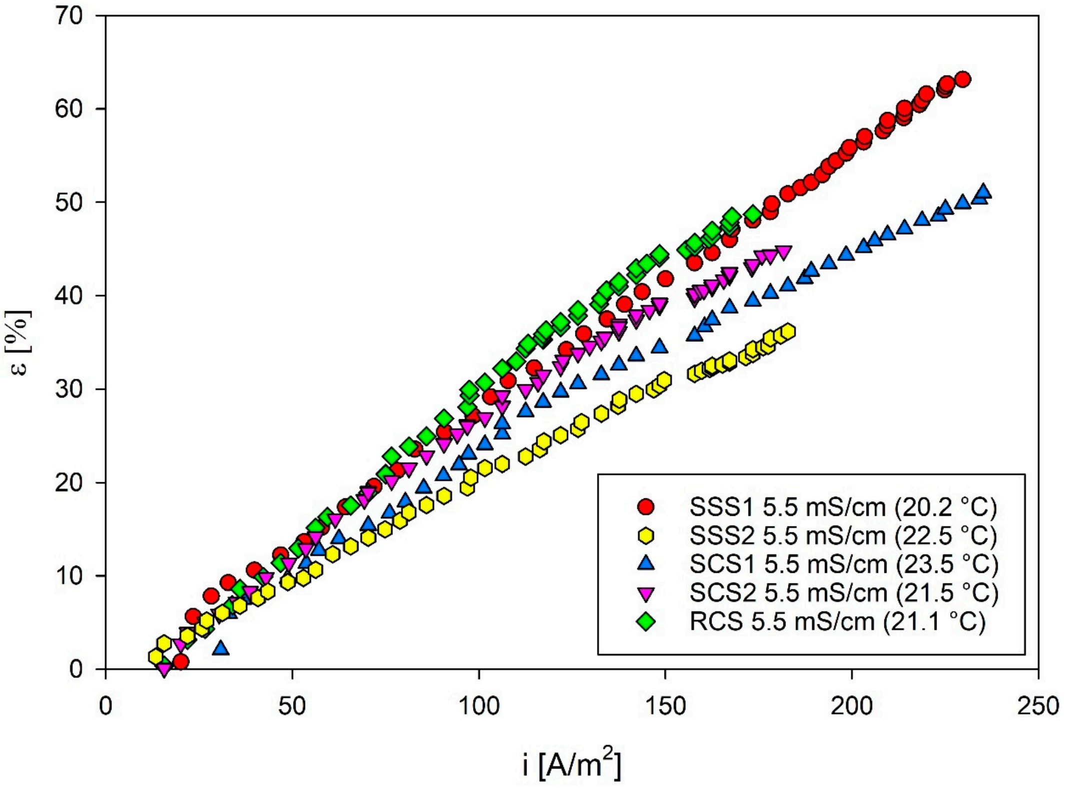

Figure S8. Removal efficiency for the salts present in the feed plotted against current density.

Fitting inverse polynomial function of a second order from SigmaPlot, determined from experimental data:

$$f(x) = y_0 - \frac{a}{x} + \frac{b}{x^2} \quad (1)$$

LCD is calculated as the function minimum in following steps:

$$f'(x) = x^{-2}(a - 2bx^{-1}) = 0 \quad (2)$$

$$LCD = x^{-1} = -\frac{a}{2b} \quad (3)$$

Table S2. Coefficients of the inverse polynomial function of a second order for estimating LCDs based on Cowan and Brown method presented in the research paper:

Solution	y_0	a	b	R^2	Std.Error
NaCl 1 mS/cm	0.5195	-0.0094	0.0002	0.7463	0.0321
NaCl 2 mS/cm	0.2962	-0.0043	5.1606E-005	0.9556	0.0080
NaCl 5.5 mS/cm	0.1677	-0.0011	4.2029E-006	0.9710	0.0026
NaCl 30.6 mS/cm	0.0300	2.0476	/	0.9881	0.0012
NaCl 48.7 mS/cm	0.0248	2.0209	/	0.9937	0.0008
SSS1 5.5 mS/cm	0.1677	-0.0011	4.2029E-006	0.9710	0.0026
SSS2 5.5 mS/cm	0.2224	-0.0016	6.7065E-006	0.8929	0.0066
SCS1 5.5 mS/cm	0.1797	-0.0008	2.2618E-006	0.9337	0.0037
SCS2 5.5 mS/cm	0.1993	-0.0013	6.3531E-006	0.9190	0.0042
RCS 5.5 mS/cm	0.1845	-0.0012	6.6920E-006	0.8343	0.0060
SCS1 1.4 mS/cm	0.6089	-0.0109	0.0001	0.7464	0.0218
SCS1 2.02 mS/cm	0.6083	-0.0111	0.0001	0.8104	0.0250
SCS1 2.93 mS/cm	0.3200	-0.0026	1.4863E-005	0.8831	0.0072
SCS2 2 mS/cm	0.5565	-0.0121	0.0002	0.8542	0.0195
SCS2 3 mS/cm	0.3445	-0.0042	4.1966E-005	0.7568	0.0145
SCS2 8 mS/cm	0.1763	-0.0011	3.1077E-006	0.9284	0.0056
SCS2 13.8 mS/cm	0.1070	-0.0004	6.0847E-007	0.8947	0.0041
RCS 2 mS/cm	0.3408	-0.0057	0.0001	0.9240	0.0157
RCS 3 mS/cm	0.2806	-0.0037	4.2574E-005	0.9215	0.0105

RCS 8 mS/cm	0.1478	-0.0008	2.7060E-006	0.9658	0.0020
RCS 12 mS/cm	0.1068	-0.0003	7.1280E-007	0.8662	0.0031
RCS 18 mS/cm	0.0877	-0.0002	3.2515E-007	0.9099	0.0028

Experimental data potted on C&B diagrams were assessed for occurrence of LCD in three ways: a–as the intersection of slopes for decreasing/increasing ED stack resistance; b–based on the current density corresponding to the minimum measured ED stack resistance; c–as the minimum of the inverse polynomial function of a second order fitted to the experimental data.

Table S3. List of solutions (SSS – 2.3.1) used in the study with corresponding LCDs defined by Cowan and Brown method in three ways (a/b/c):

**NaCl concentration [g/L]**	0.3	0.9	3.1	18.3	29.3
**Conductivity [mS/cm]**	1	2	5.5	30.6	48.7
**Average temperature T [°C]**	19.1	19.2	20.2	20.4	21.0
**Initial pH**	5.16	5.23	5.21	5.20	4.80
**LCDs [A/m²]**					
**C&B a***	28.4	56.8	175.0	/	/
**C&B b***	23.4	44.3	157.8	/	/
**C&B c***	23.5	41.7	130.9	/	/

*a/b/c – cross section of the slopes/data minimum/function minimum

Table S4. List of solutions used in the study (Chapters 2.3.1., 2.3.2., 2.3.3.) with corresponding LCDs defined by Cowan and Brown method in three ways (a/b/c):

**Solution**	**SSS1 (NaCl)**	**SSS2(Na_2SO_4)**	**SCS1**	**SCS2**	**RCS**
**Conductivity [mS/cm]**	5.5	5.5	5.5	5.5	5.5
**Average temperature [°C]**	22.6	22.5	23.5	21.5	21.1
**Initial pH**	6.31	8.49	4.59	3.24	2.25
**LCD [A/m²]**					

**C&B a***	153.6	152.4	184.2	108.8	132.5
**C&B b***	159.7	112.5	157.8	106.2	96.9
**C&B c***	148.0	119.3	176.8	102.3	89.6

*a/b/c – cross section of the slopes/data minimum/function minimum
